# Spreading of Alpha Synuclein from Glioblastoma Cells towards Astrocytes Correlates with Stem-like Properties

**DOI:** 10.3390/cancers14061417

**Published:** 2022-03-10

**Authors:** Larisa Ryskalin, Francesca Biagioni, Gabriele Morucci, Carla L. Busceti, Alessandro Frati, Stefano Puglisi-Allegra, Michela Ferrucci, Francesco Fornai

**Affiliations:** 1 Department of Translational Research and New Technologies in Medicine and Surgery, University of Pisa, Via Roma 55, 56126 Pisa, Italy; larisa.ryskalin@unipi.it (L.R.); gabriele.morucci@unipi.it (G.M.); michela.ferrucci@unipi.it (M.F.); 2 Istituto di Ricovero e Cura a Carattere Scientifico (I.R.C.C.S.) Neuromed, Via Atinense 18, 86077 Pozzilli, Italy; francesca.biagioni@neuromed.it (F.B.); carla.busceti@neuromed.it (C.L.B.); alessandro.frati@uniroma1.it (A.F.); stefano.puglisiallegra@neuromed.it (S.P.-A.); 3 Neurosurgery Division, Human Neurosciences Department, Sapienza University, 00135 Roma, Italy

**Keywords:** glioblastoma multiforme, synucleins, stem cell markers, cell clearing systems, trans-well co-culture model, mammalian target of rapamycin, transmission electron microscopy

## Abstract

**Simple Summary:**

The present study questions whether cells from glioblastoma multiforme (GBM), which overexpress α-synuclein (α-syn), may alter neighboring non-tumoral astrocyte cell lines. The occurrence of α-syn in GBM correlates with the expression of the stem cell marker nestin. When astrocytes are co-cultured with GBM cells in a trans-well apparatus the occurrence of α-syn and nestin rises remarkably. The increase in α-syn in co-cultured astrocytes is more pronounced at the plasma membrane, which mimics the placement of α-syn in GBM cells. When the mTOR inhibitor rapamycin is administered, GBM-induced expression of α-syn and nestin within co-cultured astrocytes is occluded, and morphological alterations are reverted. In the presence of rapamycin the sub-cellular placement of α-syn is modified being allocated within whorls and vacuoles instead of the plasma membrane. The effects induced by rapamycin occur both in baseline GBM cells and within astrocytes primed by co-cultured GBM cells.

**Abstract:**

Evidence has been recently provided showing that, in baseline conditions, GBM cells feature high levels of α-syn which are way in excess compared with α-syn levels measured within control astrocytes. These findings are consistent along various techniques. In fact, they are replicated by using antibody-based protein detection, such as immuno-fluorescence, immuno-peroxidase, immunoblotting and ultrastructural stoichiometry as well as by measuring α-syn transcript levels at RT-PCR. The present manuscript further questions whether such a high amount of α-syn may be induced within astrocytes, which are co-cultured with GBM cells in a trans-well system. In astrocytes co-cultured with GBM cells, α-syn overexpression is documented. Such an increase is concomitant with increased expression of the stem cell marker nestin, along with GBM-like shifting in cell morphology. This concerns general cell morphology, subcellular compartments and profuse convolutions at the plasma membrane. Transmission electron microscopy (TEM) allows us to assess the authentic amount and sub-cellular compartmentalization of α-syn and nestin within baseline GBM cells and the amount, which is induced within co-cultured astrocytes, as well as the shifting of ultrastructure, which is reminiscent of GBM cells. These phenomena are mitigated by rapamycin administration, which reverts nestin- and α-syn-related overexpression and phenotypic shifting within co-cultured astrocytes towards baseline conditions of naïve astrocytes. The present study indicates that: (i) α-syn increases in astrocyte co-cultured with GBM cells; (ii) α-syn increases in astrocytes along with the stem cell marker nestin; (iii) α-syn increases along with a GBM-like shift of cell morphology; (iv) all these changes are replicated in different GBM cell lines and are reverted by the mTOR inhibitor rapamycin. The present findings indicate that α-syn does occur in high amount within GBM cells and may transmit to neighboring astrocytes as much as a stem cell phenotype. This suggests a mode of tumor progression for GBM cells, which may transform, rather than merely substitute, surrounding tissue; such a phenomenon is sensitive to mTOR inhibition.

## 1. Introduction

GBM is a brain tumor featuring severe lethality and a high propensity to infiltrate and relapse [1,2,3]. A number of biochemical abnormalities occur in GBM [4,5,6,7,8,9,10]. Recent studies reported that a molecular complex named mechanistic target of rapamycin (mTOR) is up-regulated within GBM cells [4,11,12,13]. This complex promotes a number of biochemical effects in the cell. Among these, mTOR suppresses the autophagy machinery [14,15]. In fact, analogous to mTOR over-activity, autophagy suppression promotes stemness, infiltration, relapse, drug resistance and lethality in GBM [16,17,18,19].

The impairment of the autophagy pathway produces an altered clearance of classic autophagy substrates, including cell organelles such as mitochondria and specific autophagy-dependent proteins. Among these proteins the cellular prion protein (PrPc) and the prionoid α-synuclein (α-syn) depend crucially on autophagy machinery [20].

Thus, it is expected that these proteins accumulate within GBM as well as other tumors featuring mTOR overactivity. In the case of PrPc, this is well established [21]. Concerning α-syn, the occurrence in cancer is poorly investigated. In fact, despite being recognized as a key protein to foster neurodegeneration, α-syn in cancers was investigated only recently [22]. In particular, some expression of α-syn was found in breast and ovarian cancers [23], and some evidence was obtained in a few neuron-derived brain tumors such as medulloblastoma and neuroblastoma [24,25]. Solid evidence, instead, concerns high expression levels of α-syn in melanoma [26], a tumor, which originates from melanocytes, which analogously to astrocytes derive from neural crests. Most of the evidence concerning α-syn and cancer comes from studies in melanoma, where α-syn is present in high amount [26,27,28,29,30,31]. In melanoma, α-syn is a cancer biomarker and may promote cell proliferation [27,32].

Despite the evidence of a marked autophagy impairment, only a little evidence is available concerning the occurrence of α-syn in GBM [33]. The occurrence of α-syn in GBM cells was established by using a number of methods, including electron and light microscopy, where various antibodies’ specificity confirmed the occurrence of α-syn in GBM cells [34]. Such an increase was further confirmed by using real-time polymerase chain reaction (RT-PCR). The occurrence of α-syn was documented both concerning its overall amount and by measuring the amount of non-digested (proteinase K-resistant) α-syn.

Thus, in the present paper, we wish to question whether a high amount of α-syn, which is expressed in GBM cells compared with baseline astrocytes, may move from GBM towards astrocytes, when these cells are co-cultured. In order to assess this phenomenon a trans-well system, consisting of a microporous semipermeable membrane (pore size 0.4 μm) was used. In this system the following questions were addressed: (i) whether α-syn levels increase in astrocytes when co-cultured with GBM cells (GBM-primed astrocytes). (ii) Whether electron microscopy may provide evidence for expression of α-syn between cells and at cell-to-cell membrane interfaces. (iii) Whether the increase in α-syn in co-cultured astrocytes is concomitant with increased expression of the stem cell marker nestin. (iv) Whether the increases in α-syn and nestin are concomitant with a shifting in cell morphology and ultrastructure, mimicking GBM cells. (v) Whether rapamycin, which activates autophagy through mTOR inhibition and suppresses α-syn in GBM cells, may also occlude nestin and α-syn overexpression and produce additional phenotypic shifting within GBM-primed astrocytes.

## 2. Materials and Methods

### 2.1. Cell Cultures

The U87MG GBM cell line, obtained from Cell Bank (IRCC San Martino-IST, Genova, Italy), was grown in DMEM (Sigma Aldrich, Milan, Italy) supplemented with 10% fetal bovine serum (FBS), 1% of MEM Non-Essential Amino-Acid (MEM-NEAA), penicillin (50 IU/mL) and 100 μg streptomycin (Sigma). The GBM cell line A172 was obtained from the European Collection of Authenticated Cell Cultures (ECACC) and from Cell Bank (IRCC San Martino-IST, Genova, Italy) and maintained in Modified Eagle’s Medium (Euroclone, Milan, Italy) supplemented with 10% FBS, 2 mM L-glutamine, 100 IU/mL penicillin and 100 μg streptomycin. GBM cells were kept at 37 °C at 5% CO_2_. Normal human fetal-derived astrocytes (NHA; Lonza, CC-2565; lot #0000514417) were grown according to the manufacturer’s specifications (AGM BulletKit, Lonza, Euroclone) and kept at 37 °C at 5% CO_2_. When cultures reached approximately 80% confluence (five days after plating) sub-cultures of NHA were obtained by partial digestion with ReagentPack™ sub-culturing reagents (Lonza; CC-5034).

For immuno-cytochemistry and immuno-fluorescence experiments at light microscope 5 × 10^4^ cells were seeded on cover slips within 24-well plates within a final volume of 1 mL/well.

For electron microscopy and Western blotting, 1 × 10^6^ cells were seeded within 6-well plates with 2 mL/well.

### 2.2. Trans-Well Co-Cultures

**Figure 1:**
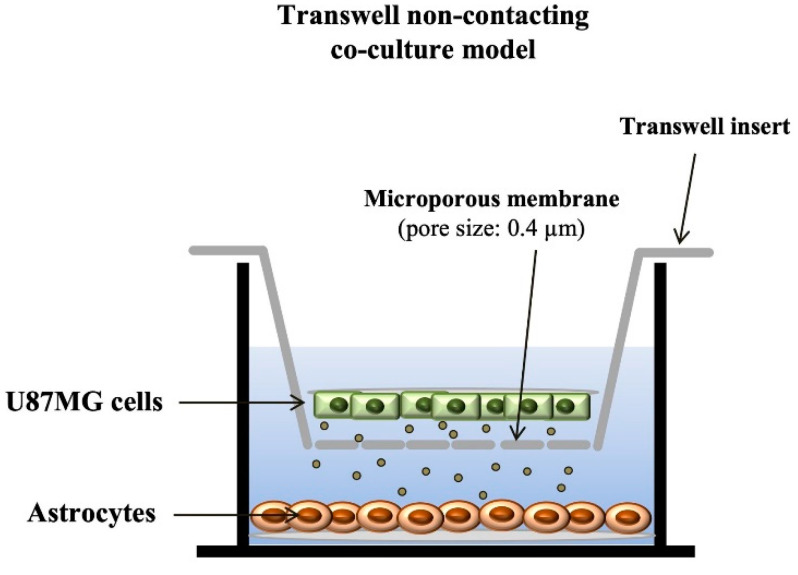
Illustration showing the trans-well co-culture system. The cartoon shows a non-contacting co-culture model of astrocytes and GBM cells. In detail, astrocytes and GBM cells are co-cultured in two different compartments, which are separated by a trans-well insert gifted of a microporous membrane. This, in turn, allows an indirect trans-cellular interaction through the diffusion of soluble factors. The culture in the lower compartment receives the treatment 24 h before the culture in the upper compartment is added. In this way the upper compartment is not directly receiving the treatment and it is mostly affected by the culture present in the lower compartment. Only cells from the upper compartment of each co-culture were counted.

The trans-well co-culture system was used for co-culturing astrocytes and U87MG cells. This system (Figure 1) consists of a trans-well insert with a microporous membrane (pore size 0.4 μm; Sarstedt, Germany), which separates the well in two different compartments (i.e., lower of the well, lower compartment, and trans-well insert, upper compartment). The system impedes direct contact between the cells growing within two different compartments, although allowing diffusion of soluble factors between cells. The culture in the lower compartment receives the treatment 24 h before the culture in the upper compartment is added. In this way, the upper compartment is not receiving directly the treatment and it is mostly affected by the culture present in the lower compartment, which was treated 24 h before. Only cells from the upper compartment of each co-culture were counted. In this way, the effects of rapamycin in the upper compartment were assessed as an indirect influence produced by the cell culture placed in the lower compartment. In the lower compartment cells were treated with either 0.01% DMSO or rapamycin 100 nM for 24 h. Afterward, cells were transferred in the lower compartment of the trans-well system to be co-cultured with either astrocytes or GBM cells placed in the upper compartment for 7 days. Thereafter, cells from the upper compartment were removed and routinely processed for immuno-peroxidase against α-syn (see Section 2.4.1 Immuno-peroxidase), immuno-fluorescence staining against α-syn and nestin (see Section 2.4.2 Immuno-fluorescence) and transmission electron microscopy (see Section 2.6. Transmission electron microscopy). Thus, cells from the upper compartment, which were counted both at light and electron microscopy correspond to the those named at first in the co-culture (for example, astrocytes were counted in co-cultures of astrocytes and GBM cells). This allows us to detect the effects of rapamycin-treated cells rather than rapamycin on the cell culture placed in the upper compartment. This was implemented in order to reduce the direct effects of rapamycin on the cells under analysis.

### 2.3. Cell Treatments

In another set of experiments, GBM cells (i.e., U87MG and A172 cell lines) were treated with various doses of rapamycin (1 nM, 10 nM, 100 nM and 1000 nM) for 24 h. A 1 mM stock solution was prepared and dissolved in culture medium containing 10% dimethyl sulfoxide (DMSO) in order to obtain the different rapamycin dilutions. Control cells were maintained in culture medium where 0.01% DMSO was added.

### 2.4. Immuno-Cytochemistry at Light Microscopy

At the end of the treatments, culture medium was removed. Cells were fixed with 4% paraformaldehyde in PBS for 15 min at room temperature and after washing with PBS they were stained with immuno-peroxidase or immuno-fluorescence.

#### 2.4.1. Immuno-Peroxidase

For immuno-peroxidase staining, fixed cells were first permeabilized by using Triton X 0.1% (Sigma) for 15 min in PBS, washed with PBS to facilitate antigen retrieval. Endogenous peroxidase activity was blocked by incubating cells in 3% hydrogen peroxide (H_2_O_2_) for 20 min. Then, cells were plunged into a blocking solution containing 10% normal goat serum (NGS) in PBS for 1 h at room temperature, followed by incubation within the primary antibody solution containing mouse anti-α-syn primary antibody (1:1000; Abcam, Cambridge, UK) or rabbit anti-CD133 primary antibody (1:1000; Abcam), 2% NGS in PBS overnight at 4 °C with. Afterwards, cells were incubated with anti-mouse or anti-rabbit biotin-conjugated secondary antibody (1:200; Vector Laboratories, Burlingame, CA, USA) for 1 h at room temperature, followed by exposure to avidin–biotin complex (ABC, Vector) for 1 h and the peroxidase substrate diaminobenzidine (DAB, Vector) for 3 min at room temperature. Finally, after dehydration in increasing alcohol solutions and clarification in xylene, cells were cover slipped with DPX mounting medium (Sigma) and observed under Nikon Eclipse 80i light microscope (Nikon, Tokyo, Japan) at 20× magnification.

#### 2.4.2. Immuno-Fluorescence

After fixing, cells were permeabilized by Triton X 0.1% for 15 min in PBS and incubated in a blocking solution (10% NGS in PBS) for 1 h at room temperature.

Single immunofluorescence staining was carried out for α-syn. The primary antibody solution containing rabbit polyclonal anti-α-syn antibody (1:100; Sigma), 2% NGS in PBS was left overnight at 4 °C. The antigen–antibody reaction was revealed by using the anti-rabbit fluorescent secondary antibody Alexa Fluor 488 (1:200; Life Technologies, Carlsbad, CA, USA), for 1 h, at room temperature. 

In another set of experiments, double immuno-fluorescence was carried out. In detail, cells were incubated overnight with a primary antibody solution containing both the mouse monoclonal anti-α-syn (1:100; Abcam) and the rabbit anti-nestin (1:100; Millipore, Temecula, CA, USA) primary antibodies. After washing out with PBS, the antigen–antibody reactions were revealed by using the anti-rabbit or the anti-mouse fluorophore-conjugated secondary antibodies (Alexa 488 or 546, Life Technologies, Carlsbad, CA, USA) diluted 1:200 in PBS at RT.

Cell nuclei were stained by incubating cells within the nuclear dye DAPI (Sigma) diluted 1:3000 in PBS for 5 min.

After washing in PBS, cells were mounted using Fluoroshield medium (Sigma).

All samples were observed using Nikon Eclipse 80i light microscope equipped with a fluorescent lamp and a digital camera connected to the NIS Elements software for image analysis (Nikon, Tokyo, Japan). DAPI-, α-syn-, and nestin-stained pictures were acquired independently and then they were merged. Final panels were prepared using Adobe Photoshop.

### 2.5. Densitometry Analysis at Light Microscopy

Densitometry analysis of α-syn- and nestin-immuno-stained cells was carried out at light microscopy, at 40× magnification by using Image J software within three distinct microscopic fields, where all cells were distinctly visualized without overlapping. Densitometry measurements were carried out within the cytosol, the nucleus, or the whole cell by two observers, who were blind to treatments. Data refer to N = 30 cells per group and are given as the mean percentage ± S.E.M. of optical density for each experimental group.

### 2.6. Transmission Electron Microscopy

Cells were centrifuged at 1000× *g* for 5 min; the supernatant was removed and the cell pellet was rinsed in PBS and incubated in a fixing solution containing 2.0% paraformaldehyde and 0.1% glutaraldehyde in 0.1 M PBS (pH 7.4) for 90 min at 4 °C. This fixing solution contains a concentration of aldehydes which minimally cover antigen epitopes while fairly preserving tissue architecture. Then, specimens were washed in PBS and post-fixed in 1% OsO_4_ for 1 h at 4 °C, dehydrated in ethanol and finally embedded in epoxy resin. Post-embedding immuno-cytochemistry procedure was carried out by using primary antibodies against α-syn and nestin (see Section 2.7).

Ultra-thin sections were counter-stained with a saturated solution of uranyl acetate and lead citrate, and were examined by using a JEOL JEM-100SX transmission electron microscope (JEOL, Tokyo, Japan). For ultrastructural morphometry, several grids containing non-serial ultrathin sections (70–90 nm thick) were analyzed, at 8000× magnification, in order to count a total of 30 cells for each experimental group.

### 2.7. Post-Embedding Immuno-Cytochemistry

Post-embedding procedure was carried out on ultra-thin sections collected on nickel grids, according to previously validated protocols, which allow minimal epitope covering, while preserving cell ultrastructure [35,36,37]. In detail, grids were incubated with OsO_4_, which binds to cell membranes and enhances the contrast of cell organelles, while preventing formation of membrane’s artifacts. Then, grids were incubated with aqueous sodium meta-periodate (NaIO_4_), for 30 min, at room temperature, to remove OsO_4_ and allowing a closer antigen–antibody contact [36].

This step is key for ultrastructural morphometry since it allows detection, localization and stoichiometric counts of protein placement within clearly defined sub-cellular compartments. In fact, high-resolution detection of colloidal gold-conjugated particles allows both the count and localization of the stoichiometric reaction, where 1 antigen molecule corresponds to 1 gold particle. In fact, gold particles appear as round electron dense spots, each one corresponding to single α-syn molecules. This provides the total quantitative assessment and compartmentalization of α-syn [38,39,40,41].

After washing in PBS, grids were incubated in a blocking solution containing 10% goat serum and 0.2% saponin for 20 min at room temperature, followed by a solution containing the rabbit primary antibody anti-α-syn (1:50; Sigma), and rabbit anti-nestin (1:150; ProteinTech, Manchester, UK) primary antibodies, with 0.2% saponin and 1% goat serum, overnight. This step was carried out in a humidified chamber, at 4 °C.

After washing in PBS, gold-conjugated secondary antibodies were used to reveal separately, each antigen–antibody solution. In detail, immuno-gold particles of either 10 nm or 20 nm mean diameter (BB International, Treviso, Italy) were used, diluted 1:20 in a solution of 0.2% saponin and 1% goat serum in PBS for 1 h, at room temperature. Then, grids were incubated with 1% glutaraldehyde, for 3 min, and extensively washed with distilled water.

### 2.8. Ultrastructural Morphometry

For ultrastructural morphometry, grids containing non-serial ultrathin sections (70–90 nm thick) were examined at TEM, at a magnification of 8000×, according to Lucocq et al., 2004 [42]. To quantify the number of immuno-gold particles, several grids were analyzed in order to count a total of 30 cells for each experimental group. In detail, within the ultrathin sections, two observers blind to treatment scanned through equally spaced parallel sweeps across the specimens starting from the corner of a squared grid. Assessment of vacuoles and measurement of immuno-gold particles were carried out according to Lenzi et al., 2016 [35]. Cell vacuoles were scored as single, double, or multiple membranes containing cytoplasmic material and electron-dense structures [43,44,45].

The following items were counted: (i) the number of α-syn immuno-gold particles within the whole cell; (ii) the number of α-syn immuno-gold particles placed within cytosol; (iii) the number of α-syn immuno-gold particles placed within the nucleus; (iv) the number of α-syn immuno-gold particles placed on the plasma membrane; (v) the number of α-syn containing vacuoles (vi) the number of α-syn immuno-gold particles placed within vacuoles. In addition: (i) the number of nestin immuno-gold particles within whole cell; (ii) the number of nestin immuno-gold particles placed within cytosol; (iii) the number of nestin immuno-gold particles within nucleus were measured.

Values are given as the mean number of immuno-gold particles or positive vacuoles per cell out of 30 cells counted.

### 2.9. Western Blotting

Cells were homogenized at 4 °C in ice-cold lysis buffer with phosphatase and protease inhibitor. One microliter of homogenates was used for protein determinations (Bradford procedure). Proteins (20 µg) were separated on sodium dodecyl sulphate polyacrylamide gels (15%) and transferred on Immuno-PVDF membranes (Bio-Rad, Milano, Italy) for 1 h. Filters were blocked 2 h in Tween-20 Tris-buffered saline (TTBS) (100 mM TrisHCl, 0.9% NaCl, 1% Tween 20, pH 7.4) containing 5% non-fat dry milk. Blots were incubated overnight at 4 °C with primary antibody mouse anti-α-syn (1:800, BD Transduction Laboratories, San Jose, CA, USA). 

For the blotting of U87MG cells, astrocytes and their supernatants, along with cell free medium, the following procedure was carried out. U87MG and astrocytes cell pellets were homogenized at 4 °C in 150 μL ice-cold lysis buffer containing phosphatase and protease inhibitor. Since the amount of proteins in the supernatant was very low compared with cell pellet, the amount of supernatant to be lysed was exceeding sixty six-fold the amount used for cell pellet. This means that the amount of supernatant being assayed for the occurrence of α-syn corresponds to the volume in which roughly 66 cell pellet occurs. This means that when comparing α-syn in the supernatant compared with the cell pellet the amount needs to be reduced sixty-six-fold in order to make a quantitative comparison. In fact, in order to obtain a comparable amount of proteins from supernatants, 10 mL of supernatant from either U87MG or astrocytes was used. The same volume (10 mL) of cell-free medium was used to assess potential occurrence of α-syn even in this compartment.

The culture medium to be blotted corresponds to the fresh medium used for either U87MG or astrocytes growth. In both cases, despite the high amount of medium, the protein content measured through the Bradford’s method was undetectable and the band of α-syn did not appear.

For the housekeeping we have used β-actin. Blots were incubated with primary mouse monoclonal anti β-actin antibody (1:50,000, Sigma) for 1 h, at room temperature. Filters were washed 3 times with TTBS buffer and then incubated for 1 h with secondary peroxidase-coupled antibodies (anti-mouse, 1:3000; Calbiochem, Milan, Italy). Immuno-staining was enhanced by chemiluminescence (GE Healthcare, Milan, Italy). Densitometry analysis was performed using ImageJ software. The data are expressed as the mean ± S.E.M. calculated from at least three blots per group.

### 2.10. Statistical Analysis

Densitometry of immuno-fluorescence and immuno-peroxidase is given as the mean percentage ± S.E.M. of optical density for each experimental group (assuming controls as 100%).

For ultrastructural morphometry, the number (or percentage) of α-syn and nestin immuno-gold particles, are given. Data are reported as the mean or the mean percentage ± S.E.M.

For Western blotting, values from optical density represent the mean ± S.E.M. of at least 3 blots per group. Western blotting of the medium culture was not quantified since the protein content measured through the Bradford’s method was undetectable.

Inferential statistics to compare groups were carried out by using one-way analysis of variance, ANOVA, with Scheffè’s post hoc analysis. Null hypothesis (H_0_) was rejected when *p* < 0.05.

## 3. Results

### 3.1. α-Syn Level Correlates with the Stem Cell Marker Nestin

**Figure 2:**
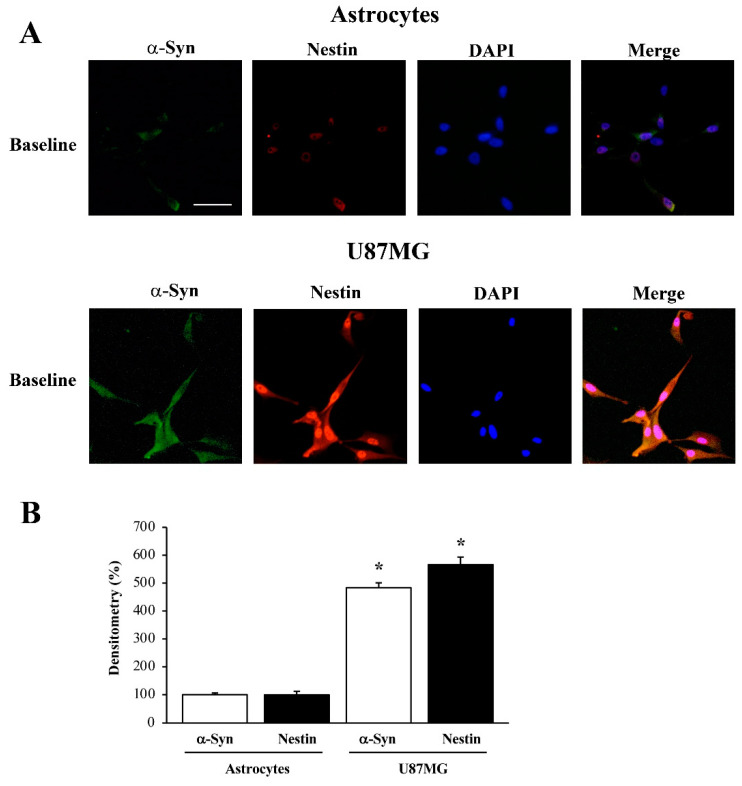
α-Syn and nestin immuno-fluorescence within astrocytes and GBM cells in baseline conditions. (**A**) Representative pictures show immuno-fluorescence and merging of α-syn (green) and nestin (red) within astrocytes (upper panels) and GBM cells (lower panels). Nuclei are stained with DAPI (blue). Densitometry of both antigens is reported in the graph (**B**). Data are given as the mean percentage ± S.E.M. of optical density for each experimental group (densitometry in astrocytes is considered as 100%) obtained from N = 30 cells per group. * *p* < 0.05 compared with astrocytes. Scale bar = 24 μm.

As shown in representative pictures of Figure 2, in baseline conditions astrocytes feature negligible levels of α-syn and nestin (Figure 2A). In contrast, in GBM cells both antigens are way in excess compared with astrocytes and such an overexpression occurs similarly for both antigens (Figure 2A). Occurrence of α-syn and nestin in astrocytes and GBM cells is expressed by fluorescence densitometry, and it is reported in the graph of Figure 2B. It is remarkable that there is a higher expression of nestin parallels than of α-syn when GBM cells are compared with astrocytes. In representative pictures at light microscopy, the increase in nestin seems to be more focused in the nucleus compared with the increase in α-syn, which is quite widespread. This is evidenced by the merging between DAPI and nestin, which shows pink areas mostly at nuclear level, while the yellowish/brownish merging of nestin and α-syn immuno-staining prevails in the extra-nuclear regions (Figure 2A).

### 3.2. α-Syn Levels Are Increased within Astrocytes Co-Cultured with GBM Cells

**Figure 3:**
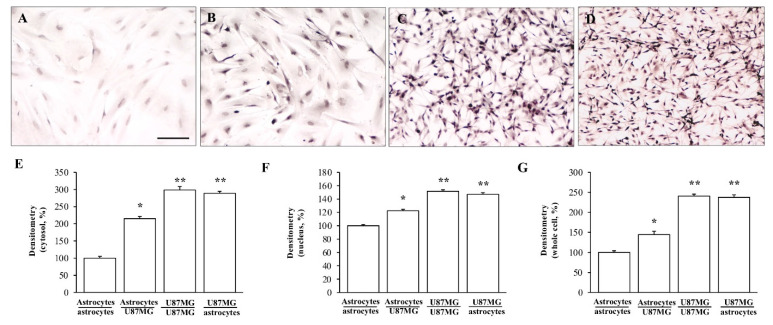
α-Syn immuno-cytochemistry in co-cultures of astrocytes and GBM cells. Representative pictures of α-syn immuno-cytochemistry within astrocytes co-cultured with astrocytes (**A**), astrocytes co-cultured with GBM cells (**B**), GBM cells co-cultured with GBM cells (**C**), and GBM cells co-cultured with astrocytes (**D**). Graphs report densitometry of α-syn staining within the cytosol (**E**), nucleus (**F**), and whole cell (**G**). Measurements refer to the cells, which are named at first in the co-culture. Data are given as the mean percentage ± S.E.M. of optical density for each experimental group (assuming astrocytes co-cultured with astrocytes as 100%) obtained from N = 30 cells per group. * *p* < 0.05 compared with astrocytes co-cultured with astrocytes. ** *p* < 0.05 compared with astrocytes co-cultured with GBM cells. Scale bar = 35.4 μm.

As reported in representative pictures of Figure 3, immuno-peroxidase for α-syn in the trans-well co-culture system shows a marked difference in α-syn levels within astrocytes, which depends on whether these cells are co-cultured either with baseline astrocytes or GBM cells. In fact, α-syn immuno-staining continues to be barely detectable when astrocytes are co-cultured with astrocytes (Figure 3A) compared with a remarkable α-syn immuno-reactivity, which occurs when astrocytes are co-cultured with GBM cells (Figure 3B). In this experimental condition α-syn immuno-staining within astrocytes reaches up to a level, which is comparable to GBM cells, either co-cultured with similar GBM cells (Figure 3C) or with astrocytes (Figure 3D). One needs to consider that the difference in α-syn levels between GBM cells co-cultured with GBM cells and GBM cells co-cultured with astrocytes is negligible, and it is comparable with a plain single culture of GBM cells. These representative pictures suggest that the increase in α-syn, which occurs when astrocytes are primed by co-cultured GBM cells, is much more evident in elongating cell poles, as it is for GBM cells. Accordingly, these GBM-primed astrocytes develop a fusiform shape with a thinning of the nuclear area and cell shape as it occurs typically for GBM cells.

The various placements of α-syn in co-cultures of astrocytes and GBM cells within cytosol, nucleus, and whole cell is reported in the graphs of Figure 3E, Figure 3F, and Figure 3G, respectively.

### 3.3. Amount and Compartmentalization of α-Syn Measured at Ultrastructural Stochiometry

**Figure 4:**
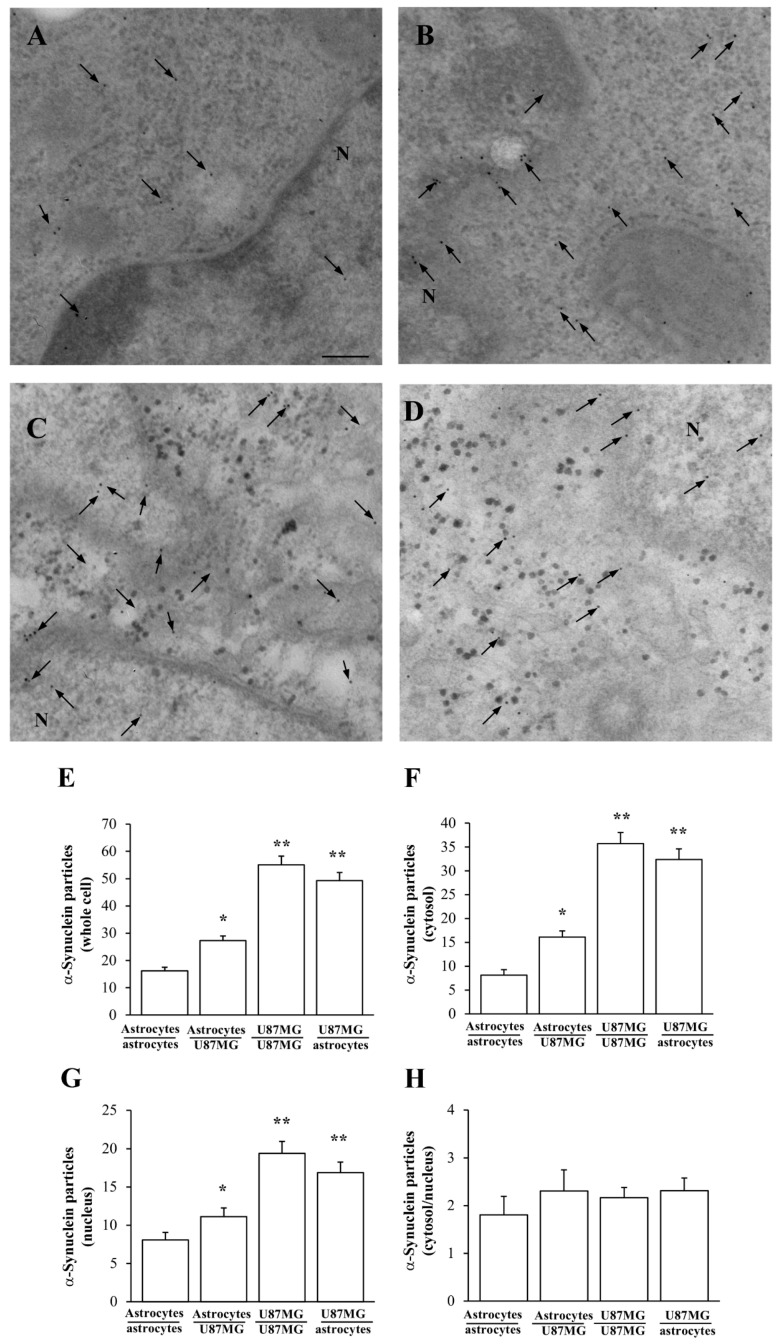
α-Syn increases within astrocytes co-cultured with GBM cells. Representative micrographs show α-syn immuno-gold particles within cytoplasm and nucleus (black arrows) of astrocytes (also representative of astrocytes co-cultured with astrocytes) (**A**); astrocytes co-cultured with GBM cells (**B**); GBM cells (similar to GBM cells co-cultured with other GBM cells (**C**), which replicates findings on GBM cells co-cultured with astrocytes (**D**). N = nucleus. The cell population under examination is the first one listed in the co-culture (i.e., astrocytes when examining a co-culture of astrocyte with GBM; or GBM when examining a co-culture of GBM with astrocytes). Such an explanation is needed in order to specify, which cell culture is specifically observed and quantified for each measurement. Graphs report the number of α-syn immuno-gold-particles within whole cell (**E**), cytosol (**F**), and nucleus (**G**). The ratio of α-syn immuno-gold particles within cytosol vs. nucleus is reported in (**H**). Data are given as the mean *±* S.E.M. from N = 30 cells per each experimental group. * *p* < 0.05 compared with astrocytes co-cultured with astrocytes; ** *p* < 0.05 compared with astrocytes co-cultured with GBM cells. Scale bar = 1 μm.

As shown in the representative picture of Figure 4A, the occurrence of α-syn immuno-gold particles (black arrows) is scarce within astrocytes (this occurs similarly, in baseline conditions and when they are co-cultured with astrocytes). In contrast, high levels of α-syn occur within astrocytes when these cells are co-cultured with GBM cells (Figure 4B). In this latter experimental condition, α-syn approaches the amount observed in baseline GBM cells (similar to GBM co-cultured with GBM cells, as evident in representative picture of Figure 4C). When GBM cells are co-cultured with astrocytes, α-syn immuno-gold particles are similarly high (Figure 4D). Thus, exposure of astrocytes to GBM cells rises α-syn in astrocytes up to levels approaching those detected within GBM cells.

**Figure 5:**
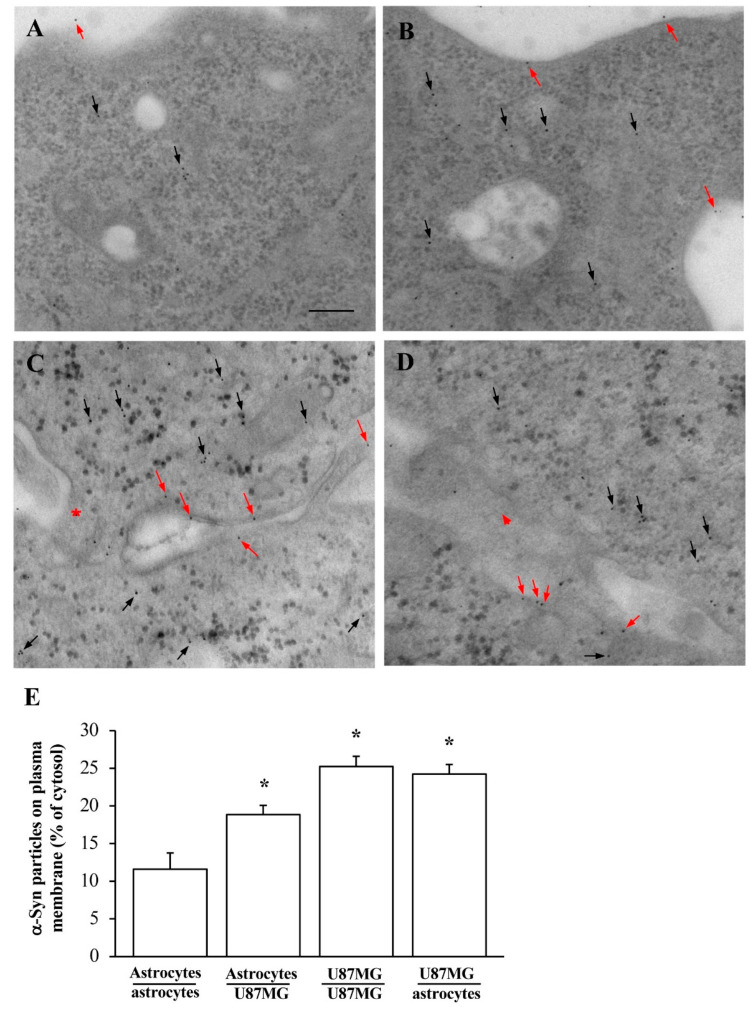
Increase and misplacement of α-syn in astrocytes exposed to GBM cells. Representative micrographs show astrocytes co-cultured with astrocytes (**A**); astrocytes co-cultured with GBM cells (**B**); GBM cells co-cultured with GBM cells (**C**), and GBM cells co-cultured with astrocytes (**D**). These representative micrographs show that plasma membrane of baseline astrocytes is quite plain. At this level, α-syn is scarcely present as shown by red arrows, while black arrows point cytosolic α-syn. In contrast, α-syn is often found at the plasma membrane when astrocytes are co-cultured with GBM cells (**B**) or within GBM cells in each conditions (**C**,**D**). In these cells α-syn is shown within cytoplasmic bridges (red asterisk) and/or in the narrow space between two cells (red arrowhead). It is remarkable that when astrocytes are co-cultured with GBM cells their plasma membranes are no longer plain, rather developing convolutions shaping bridges and tunneling analogous to GBM cells. Accordingly, α-syn is mostly placed at these membrane elongations. Graph (**E**) reports the percentage of α-syn immuno-gold particles counted at the plasma membrane compared with those measured in the cytosol. The amount of α-syn molecules placed at the plasma membrane (often on cell processes) is higher in GBM cells compared with astrocytes (including co-culture of astrocytes with astrocytes). Remarkably, α-syn is increased within astrocytes following when co-cultured with GBM cells. Data are given as the mean percentage *±* S.E.M. of immuno-gold particles at plasma membrane for each experimental group (assuming cytosol as 100%) obtained from N = 30 cells per group. * *p* < 0.05 compared with astrocytes co-cultured with astrocytes. Scale bar = 0.12 μm.

This is counted by applying ultrastructural stoichiometry of the protein and it is reported in the graphs of Figure 4. In detail, when counting α-syn immuno-gold particles within astrocytes co-cultured with GBM cells, the amount of α-syn in the whole cell is significantly higher, which markedly contrasts with the low amount measured within astrocytes co-cultured with astrocytes (Figure 4E). As reported in representative picture of Figure 5A, the distribution of α-syn within the plasma membrane within astrocytes (either alone or in co-culture with other astrocytes) is scarce (red arrows) compared with GBM cells (either alone, or co-cultured with other GBM cells). Instead, when astrocytes are co-cultured with GBM cells the amount of α-syn on the plasma membrane increases (Figure 5B). Such an increase mimics what happens within GBM cells; this is shown both within GBM co-cultured with GBM cells (Figure 5C), and in GBM cells co-cultured with astrocytes (Figure 5D). The cell population under examination for counting purposes represents the first one listed in the co-culture (i.e., astrocytes when examining a co-culture of astrocyte with GBM; or GBM when examining a co-culture of GBM with astrocytes). This clarification is needed in order to emphasize which cell culture is specifically observed and quantified.

When examined at TEM, the amount of α-syn which is present at the plasma membrane of astrocytes is scarce, and remarkably it occurs on a plain perimeter of plasma membrane, lying on non-specific, plain, membrane structures. In contrast, in GBM cells the plasma membrane is much more complex, being enriched with cell-elongating poles due to processes known to participate to cell-to-cell transmission such as tunneling nanotubes (as shown in Figure 5C) or other membrane structures participating in trogocytosis or exosome-like vesicles (as shown in Figure 5D). It is remarkable that, when astrocytes are co-cultured with GBM cells morphological and ultrastructural GBM-like shifting takes place, which includes plasma membrane developing cell-to-cell communicating membranous structures similarly to that described in GBM cells (already evident at plain electron microscopy). As shown in the graph of Figure 5E, a few α-syn particles occur at the plasma membrane within astrocytes in baseline conditions (equivalent to co-cultures with other astrocytes). In contrast, when astrocytes are co-cultured with GBM cells α-syn particles at the plasma membrane increase remarkably, and they exceed those measured in baseline astrocytes two-fold. This mimics what measured at the membranes of GBM cells (including GBM cells cultured with other GBM cells, Figure 5). These areas enriched with α-syn correspond to cell-to-cell elongating structures.

### 3.4. When Astrocytes Are Co-Cultured with GBM Cells the Increase in α-Syn Accompanies the Increase in Nestin

**Figure 6:**
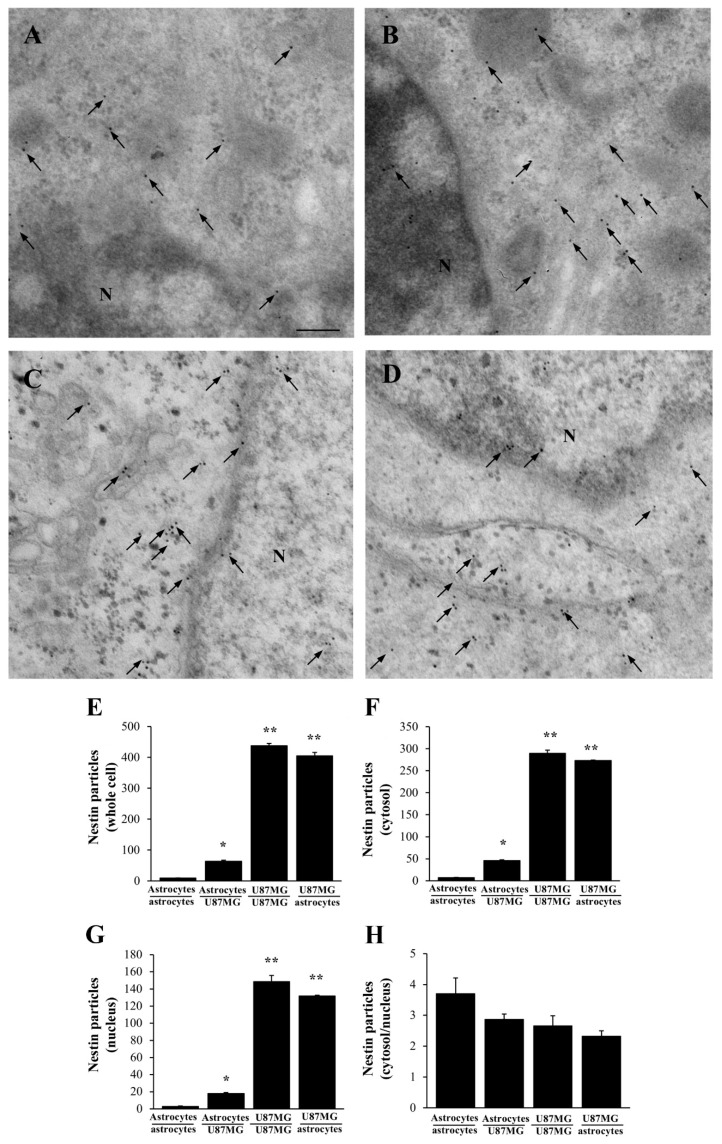
Nestin increases within astrocytes co-cultured with GBM cells. Representative micrographs show nestin immuno-gold particles (black arrows) within both cytosol and nucleus of astrocytes (including astrocytes co-cultured with astrocytes, (**A**). These particles increase within astrocytes co-cultured with GBM cells (**B**), and they are very abundant both within GBM cells co-cultured with other GBM cells (**C**), and within GBM cells co-cultured with astrocytes (**D**). N = nucleus. Graphs report the number of nestin immuno-gold-particles within whole cell (**E**), cytosol (**F**), and nucleus (**G**). The ratio of nestin immuno-gold particles between cytosol and nucleus is also counted (**H**). Nestin increases within astrocytes co-cultured with GBM cells. However, such an amount remains much lower both in whole cell, cytosol and nucleus compared with the amount counted within GBM cells. Data are given as the mean ± S.E.M. immuno-gold particles per cell obtained from N = 30 cells per each experimental group. * *p* < 0.05 compared with naïve astrocytes (including astrocytes co-cultured with astrocytes); ** *p* < 0.05 compared with astrocytes co-cultured with GBM cells. Scale bar = 0.12 μm.

In order to document whether the increase in α-syn is associated with stem-cell-like staining, detection of nestin was carried out in the very same experimental conditions. Representative micrographs of Figure 6 show that nestin is scarce within astrocytes (including those astrocytes co-cultured with astrocytes, Figure 6A). A higher amount occurs within astrocytes following co-culture with GBM cells (Figure 6B). The amount of nestin is very high within GBM cells (either alone, Figure 6C, or when co-cultured with astrocytes, Figure 6D). These findings replicate those obtained for α-syn. However, one should consider that stoichiometry of nestin indicates a net protein amount, which exceeds roughly the net levels of α-syn by roughly ten-fold. The increase in nestin within astrocytes in co-cultures with GBM cells does occur without changes concerning sub-cellular placement. In fact, neither in astrocytes nor in GBM cells does nestin match the sub-cellular placement of α-syn. In GBM cells, occurrence of nestin is rather spread in the cytosol and mostly the nucleus, rather than being concentrated at plasma membrane processes; within astrocytes the negligible amount of nestin is similarly scattered in the whole cell. This is confirmed by counts at ultrastructural stoichiometry as reported in the graphs of Figure 6. In detail, when counting nestin immuno-gold particles within the whole cell, in astrocytes only a negligible amount is detected (Figure 6E). This amount increases significantly following the co-culture with GBM cells. However, such an increase remains way below the amount of nestin counted within GBM cells, either alone or when bordering astrocytes in co-cultures. The increase in nestin within GBM-primed astrocytes occurs both within the cytosol (graph Figure 6F) and nucleus (graph Figure 6G). In fact, no difference is significantly detected between groups, concerning the ratio of nestin immuno-gold particles within cytosol vs. nucleus (graph of Figure 6H).

### 3.5. Rapamycin Dose-Dependently Suppresses α-Syn and Nestin Immuno-Fluorescence

Since it has been demonstrated that rapamycin dose-dependently mitigates over-expression of α-syn in GBM cells cultured alone [34]; the present manuscript also questions whether such a mitigation induced by rapamycin occurs for the concomitant expression of α-syn and the stem cell marker nestin. At first, this was answered in GBM cells in baseline conditions, and it was extended to astrocytes co-cultured with GBM cells, where nestin expression increases.

**Figure 7:**
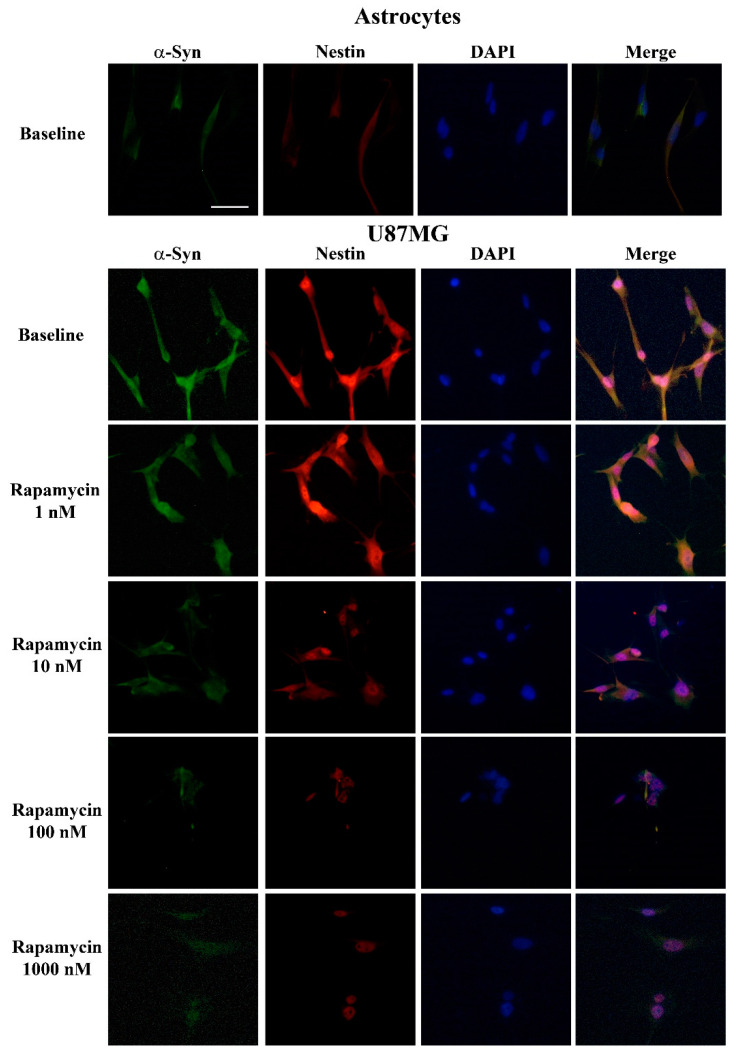
Rapamycin dose-dependently reduces α-syn and nestin immuno-fluorescence in GBM cells to an amount which is similar to baseline astrocytes (representative pictures). Representative pictures show immuno-fluorescence for α-syn (green), and nestin (red) within astrocytes and GBM cells, both in baseline conditions and following rapamycin administration. Each cell nucleus is visualized by using DAPI (blue), merging between antigens is shown as pink. Scale bar = 24 μm.

**Figure 8:**
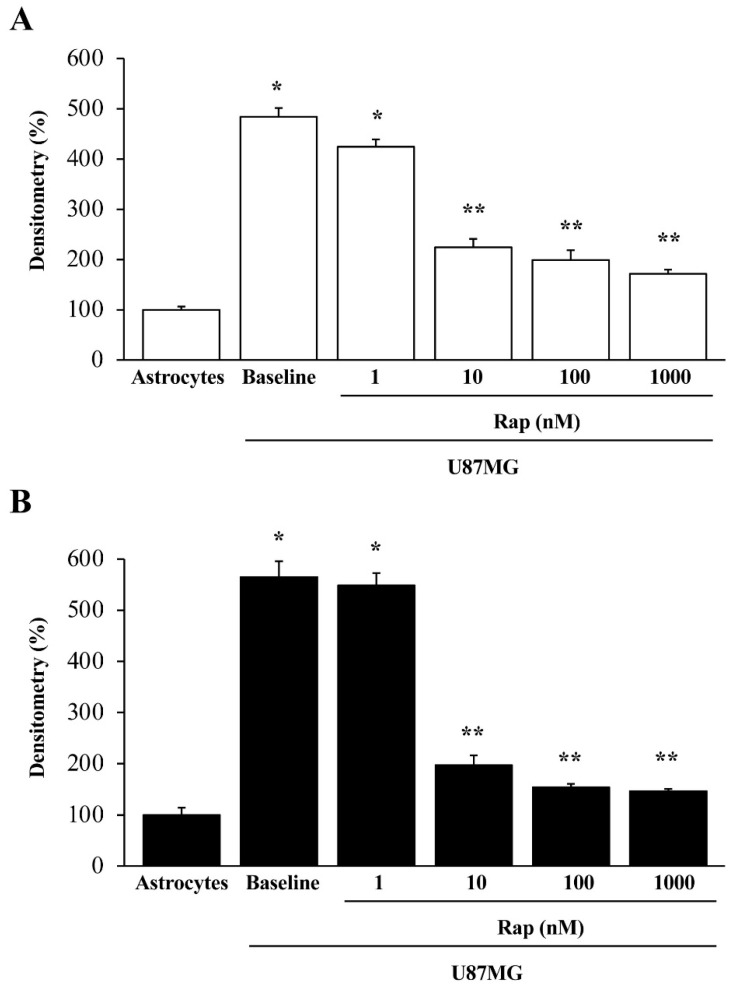
Rapamycin dose-dependently reduces α-syn and nestin immuno-fluorescence in GBM cells to an amount, which is similar to baseline astrocytes. Graphs report densitometry for α-syn (**A**) and nestin (**B**) immuno-fluorescence within baseline astrocytes and GBM cells, both in baseline conditions (baseline) and following various doses of rapamycin (Rap). Data are given as the mean percentage ± S.E.M. of optical density for each experimental group (assuming astrocytes as 100%) obtained from N = 30 cells per group. * *p* < 0.05 compared with astrocytes. ** *p* < 0.05 compared with astrocytes and GBM cells (U87MG) in baseline conditions.

As reported in representative pictures of Figure 7, while a faint immuno-fluorescent signal is detected concerning both α-syn and nestin in control astrocytes, these very same antigens were intensely immuno-fluorescent in baseline GBM cells. Twenty-four hours following rapamycin administration (ranging from 1 nM up to 1000 nM) both nestin and α-syn immuno-fluorescence in GBM cells are suppressed; this is evident already at the dose of 10 nM rapamycin, for both U87MG and A172 GBM cells (Figure 7, Supplementary Figures S1–S4). The representative images showing the effects of rapamycin in astrocytes are not reported here, due to a negligible immuno-fluorescence, which is present in these cells in baseline conditions making the effects of rapamycin non-detectable. It is remarkable that, although the net amount is ten-fold different, the massive suppression of α-syn expression in GBM cells occurs similarly to that of the stem cell marker nestin when rapamycin is administered at therapeutic doses. The amount of both nestin and α-syn immunofluorescence in GBM cells following rapamycin is comparable to that shown in baseline astrocytes (compare representative pictures of Figure 7). This amount is negligible and increasing the dose of rapamycin above 100 nM (exceeding the therapeutic range) does not produce any further decrease. These representative images are consistent with densitometry measurement of both α-syn and nestin (graphs of Figure 8A,B, respectively). Similarly to nestin, rapamycin reduces the immunostaining for the stem cell marker CD133 in both U87MG and A172 GBM cells (Supplementary Figures S5 and S6, respectively).

### 3.6. Overexpression of α-Syn and Nestin in GBM-Co-Cultured Astrocytes Is Occluded by Rapamycin

**Figure 9:**
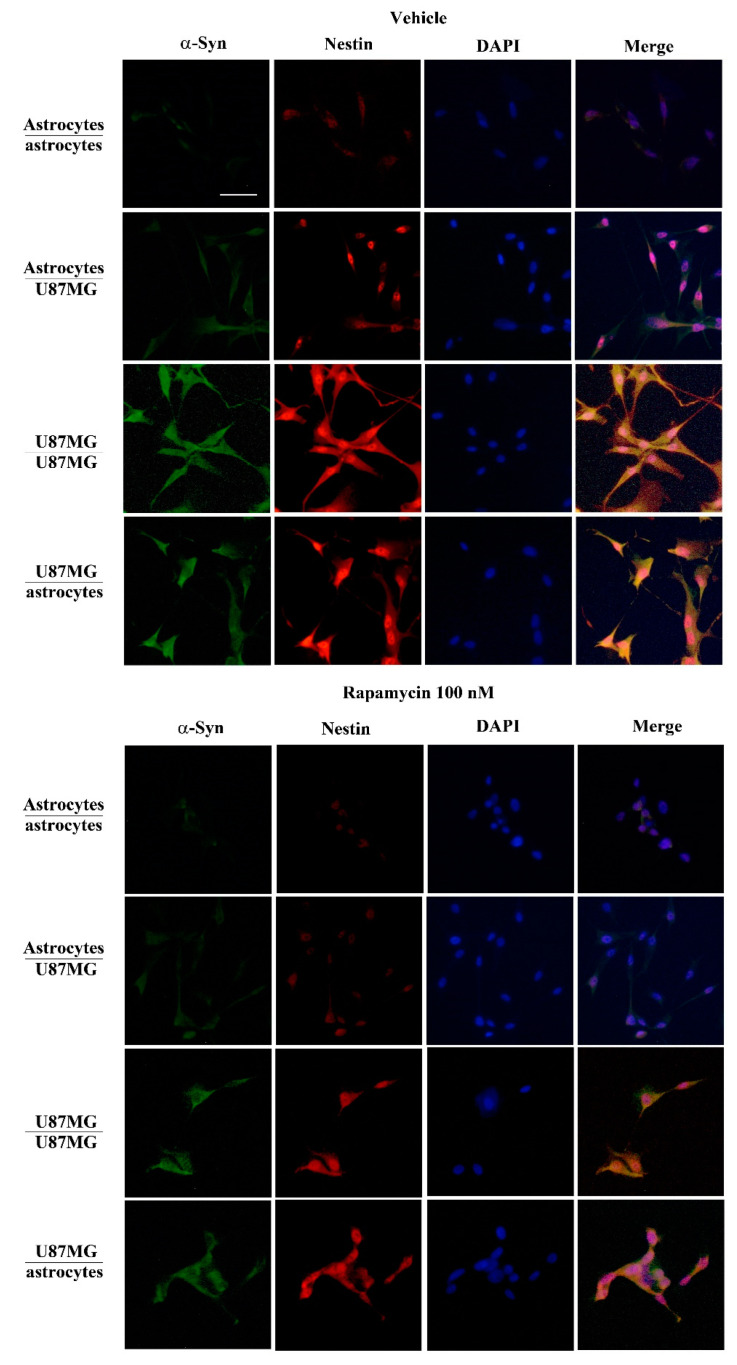
α-Syn parallels nestin expression in co-cultures of astrocytes and GBM cells with or without rapamycin (100 nM). Representative pictures show immuno-fluorescence for α-syn (green), nestin (red) and nucleus DAPI fluorescence (blue) and their merge (pink), within co-cultures of astrocytes and GBM cells in baseline conditions (vehicle) and following rapamycin administration (Rapamycin 100 nM). Scale bar = 28 μm.

**Figure 10:**
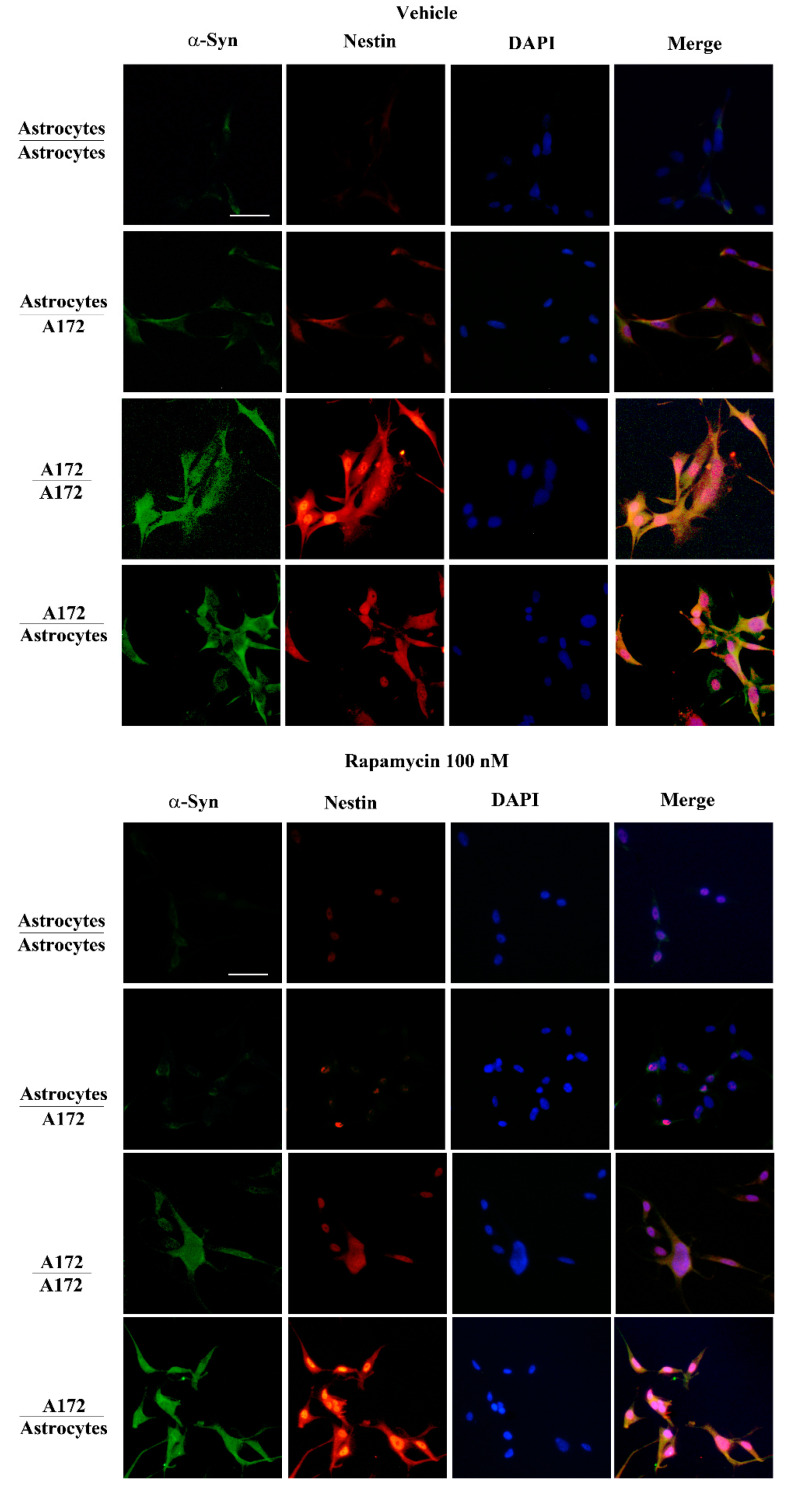
α-Syn parallels nestin expression in co-cultures of astrocytes and A172 cells with or without rapamycin (100 nM). Representative pictures show immuno-fluorescence for α-syn (green), nestin (red) and nucleus DAPI fluorescence (blue) and their merge, within co-cultures of astrocytes and A172 cells in baseline conditions (vehicle) and following rapamycin administration (Rapamycin 100 nM). Scale bar = 30 μm.

**Figure 11:**
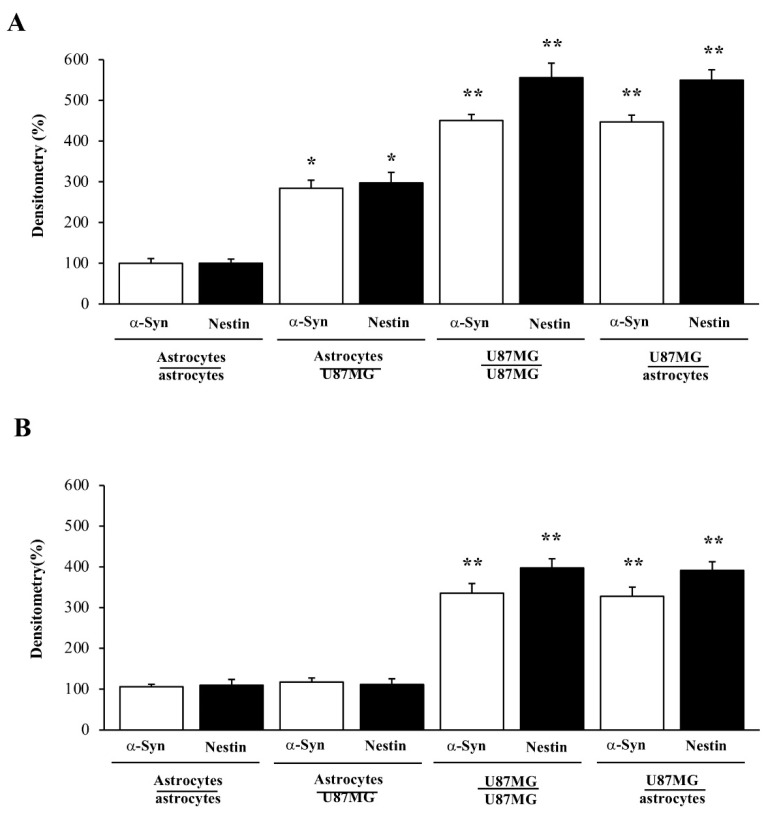
Densitometry of α-syn and nestin expression in co-cultures of astrocytes and GBM cells in baseline conditions and following rapamycin administration. Graphs report densitometry for α-syn and nestin immuno-fluorescence in co-cultures of astrocytes and GBM cells, both in baseline conditions (**A**), and following 100 nM rapamycin administration (**B**). These data are obtained from astrocytes co-cultured with astrocytes, astrocytes co-cultured with GBM cells, GBM cells co-cultured with GBM cells, and GBM cells co-cultured with astrocytes. Data are given as the mean percentage ± S.E.M. of optical density for each experimental group (assuming astrocytes co-cultured with astrocytes as 100%) obtained from N = 30 cells per group. * *p* < 0.05 compared with astrocytes co-cultured with astrocytes; ** *p* < 0.05 compared with astrocytes co-cultured with GBM cells.

**Figure 12:**
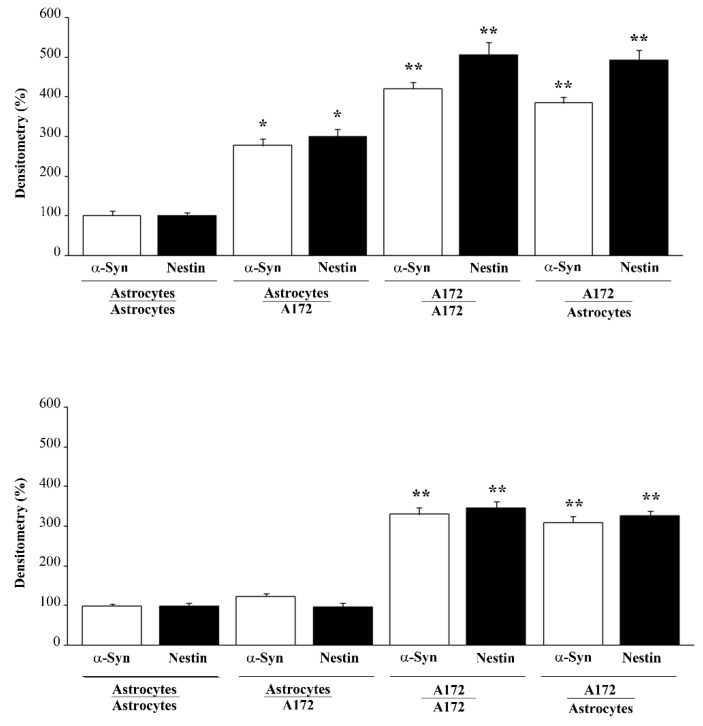
Densitometry of α-syn and nestin in co-cultures of astrocytes and A172 cells in baseline conditions and following rapamycin administration. Graphs report densitometry for α-syn and nestin immuno-fluorescence in co-cultures of astrocytes and A172 cells, both in baseline conditions (**A**) and following 100 nM rapamycin administration (**B**). These data are obtained from astrocytes co-cultured with astrocytes, astrocytes co-cultured with A172 cells, A172 cells co-cultured with A172 cells, and A172 cells co-cultured with astrocytes. Data are given as the mean percentage+S.E.M. of optical density for each experi-mental group (assuming astrocytes co-cultured with astrocytes as 100%) obtained from N = 30 cells per group. * *p* < 0.05 compared with astrocytes co-cultured with astrocytes; ** *p* < 0.05 compared with astrocytes co-cultured with A172 cells.

As expected, in the trans-well system, co-cultured GBM cells increase the amount of both α-syn and nestin within astrocytes, which otherwise possess negligible amounts of both proteins (Figure 9, upper panels, and Figure 10, upper panels). Since expression of both α-syn and nestin is suppressed dose-dependently within GBM cells (Figure 7, Figure 8 and Figure 10 and Supplementary Figures S1–S4) the present piece of data answers the seminal question of whether conditional overexpression of both proteins in GBM-primed astrocytes could be occluded by exposure to doses of rapamycin at therapeutic range (up to 100 nM). In fact, as shown in Figure 11A and Figure 12A, both α-syn and nestin significantly increase in astrocytes co-cultured with GBM cells compared with baseline astrocytes, while the exposure to rapamycin at the dose of 100 nM suppresses such an effect (Figure 11B and Figure 12B). Again, it is remarkable that rapamycin-induced suppression of α-syn level within astrocytes co-cultured with GBM cells is induced concomitantly with the suppression of nestin level. Rapamycin administered to GBM-primed astrocytes brings down α-syn and nestin levels to that measured in baseline astrocytes (Figure 11B and Figure 12B). In fact, the slight signal, which is detectable within astrocytes co-cultured with GBM cells following rapamycin is comparable with that observed within astrocytes co-cultured with astrocytes. This markedly contrasts with the intense immuno-fluorescence of both α-syn and nestin observed within GBM cells co-cultured with either GBM cells or astrocytes (Figure 11A and Figure 12A). Again, exposure to rapamycin suppresses α-syn and nestin immuno-fluorescence within GBM cells, both when co-cultured with themselves and with astrocytes (Figure 11B and Figure 12B). Immuno-staining for the stem cell marker CD133 overlaps nestin immunostaining in co-cultures of astrocytes and U87MG (Supplementary Figure S7) or A172 (Supplementary Figure S8) cells, both in baseline conditions and following rapamycin treatment (Supplementary Figures S7 and S8).

**Figure 13:**
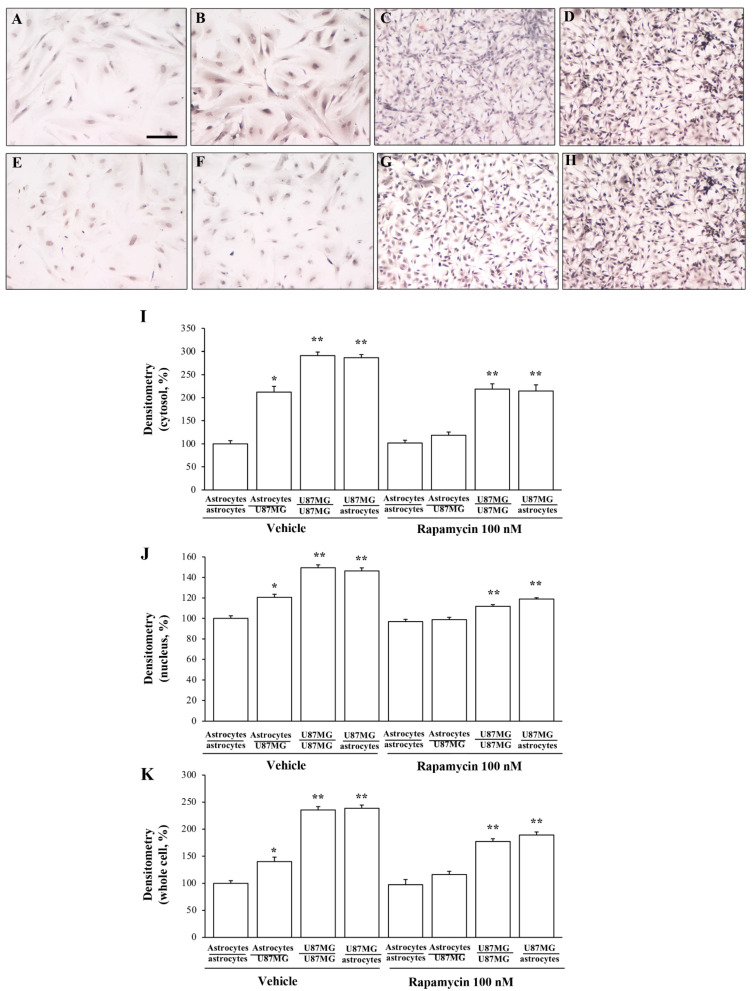
α-Syn immuno-cytochemistry in co-cultures of astrocytes and GBM cells, both in baseline conditions and following rapamycin administration. Upper panels report representative pictures of α-syn immuno-cytochemistry within astrocytes co-cultured with astrocytes (**A**), astrocytes co-cultured with GBM cells (**B**), GBM cells co-cultured with GBM cells (**C**), and GBM cells co-cultured with astrocytes (**D**) in baseline conditions. Lower panels report representative pictures of α-syn immuno-cytochemistry within astrocytes co-cultured with astrocytes treated with rapamycin (**E**), astrocytes co-cultured with GBM cells treated with rapamycin (**F**), GBM cells co-cultured with GBM cells treated with rapamycin (**G**), and GBM cells co-cultured with astrocytes treated with rapamycin (**H**). Graphs show that rapamycin reduces α-syn levels within astrocytes co-cultured with GBM cells. Graphs report densitometry of α-syn immuno-staining within the cytosol (**I**), nucleus (**J**), and whole cell (**K**) in co-cultures of astrocytes and GBM cells, both in baseline conditions (vehicle) and following rapamycin administration. Data are given as the mean percentage ± S.E.M. of optical density for each experimental group (assuming astrocytes co-cultured with astrocytes as 100%) obtained from N = 30 cells per group. * *p* < 0.05 compared with astrocytes co-cultured with astrocytes; ** *p* < 0.05 compared with astrocytes co-cultured with GBM cells. Scale bar = 35.4 μm.

Similar to immuno-fluorescence, immuno-peroxidase for α-syn decreases when the trans-well co-cultures of astrocytes are exposed to rapamycin (Figure 13).

The differences in α-syn levels in the trans-well co-cultures of astrocytes and GBM cells within cytosol, nucleus, and whole cell is measured by densitometry, and it is reported in the graphs of Figure 13I, Figure 13J and Figure 13K, respectively. Apart from different intensity in baseline conditions, the decrease in α-syn immuno-staining induced by rapamycin is comparable for cytosol and nucleus, and consistently for the whole cell. The effects of rapamycin in U87MG cells co-cultured with other U87MG cells or astrocytes are significant, although less pronounced compared with the effects obtained by administering rapamycin directly to a single U87MG cell culture. This is likely to depend on the fact that antigens (α-syn and nestin) are counted in those U87MG cells, which are added in the trans-well at 24 h following rapamycin administration to the other cell culture (either U87MG or astrocytes). This may render the effects of rapamycin as slight, due to a dose reduction and a significant time interval (7 days) compared with the effects observed within cells, which received directly rapamycin (100 nM, for 24 h).

### 3.7. Ultrastructural Stoichiometry Quantifies Rapamycin-Induced Nestin Decrease in Astrocytes Co-Cultured with GBM Cells

**Figure 14:**
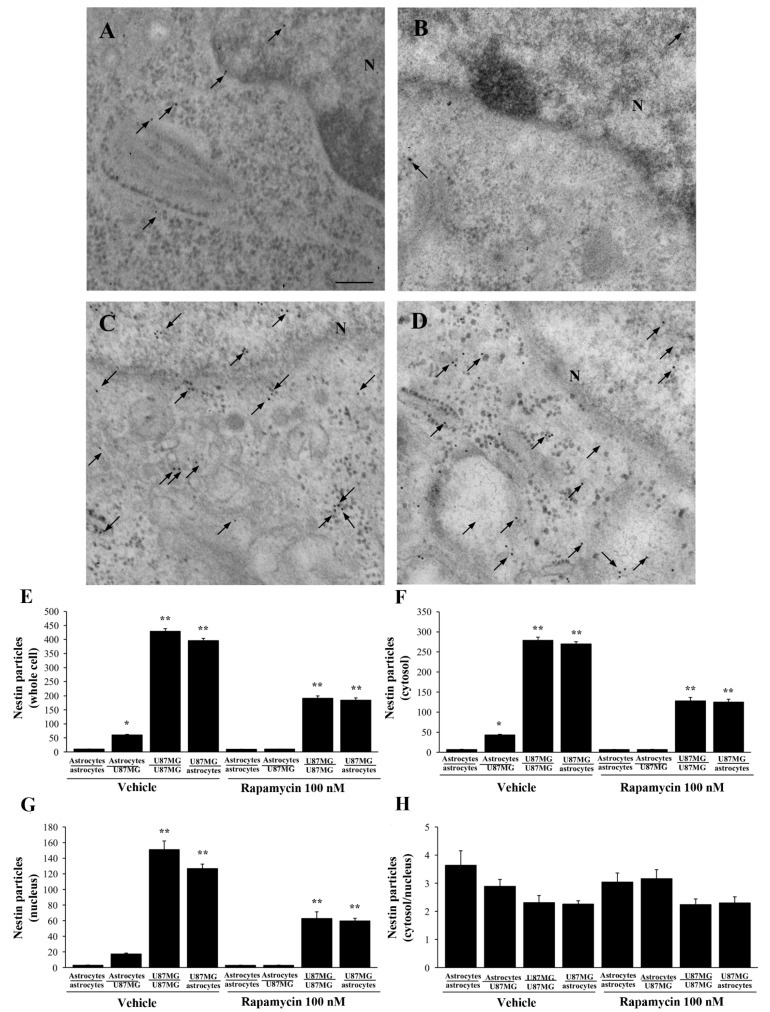
Rapamycin reduces nestin immuno-gold particles within astrocytes co-cultured with GBM cells. Representative micrographs show nestin immuno-gold particles within both the cytosol and nucleus (black arrows) of astrocytes co-cultured with astrocytes treated with rapamycin (**A**), astrocytes co-cultured with GBM cells treated with rapamycin (**B**), GBM cells co-cultured with GBM cells treated with rapamycin (**C**), and GBM cells co-cultured with astrocytes treated with rapamycin (**D**). N = nucleus. Ultrastructural stoichiometry of nestin immuno-gold in co-cultures of astrocytes and GBM cells following rapamycin administration is reported in the graphs. In detail, graphs report the number of nestin immuno-gold-particles within whole cell (**E**), cytosol (**F**), and nucleus (**G**). The ratio of nestin immuno-gold particles within cytosol vs. nucleus is reported in (**H**). Data are given as the mean *±* S.E.M. from N = 30 cells per each experimental group. * *p* < 0.05 compared with astrocytes co-cultured with astrocytes, ** *p* < 0.05 compared with astrocytes co-cultured with GBM cells. Scale bar = 0.12 μm.

Data obtained at light microscopy are further detailed at ultrastructural stoichiometry for both nestin and α-syn. As expected, representative micrographs of Figure 14 show that following rapamycin the amount of nestin is very low within astrocytes co-cultured with astrocytes (Figure 14A) compared with GBM cells co-cultured with GBM cells (Figure 14B). A remarkable decrease in nestin immuno-gold particles is observed following rapamycin in astrocytes co-cultured with GBM cells, which in turn become similar to control astrocytes (Figure 14C). At the same time, rapamycin significantly affects the amount of nestin immuno-gold particles, which occurs within GBM cells co-cultured with astrocytes (Figure 14D). These cells feature a decrease of protein amount, which is comparable with GBM cells co-cultured with GBM cells (Figure 14B). This is confirmed by ultrastructural stoichiometry of the protein reported in the graphs of Figure 14, which indicate that the high amount of nestin, which occurs within astrocytes primed with GBM cells in co-culture, is suppressed by rapamycin. In detail, when counting nestin immuno-gold particles within the whole cell (Figure 14E), cytosol (Figure 14F), nucleus (Figure 14G), and the relative intracellular distributions of the protein across the cytosol and nucleus (Figure 14H), rapamycin-induced suppression of the protein within astrocytes co-cultured with GBM cells is similar within all compartments. 

### 3.8. Ultrastructural Stoichiometry Quantifies Rapamycin-Induced α-Syn Decrease and Compartmentalization within Astrocytes Co-Cultured with GBM Cells

**Figure 15:**
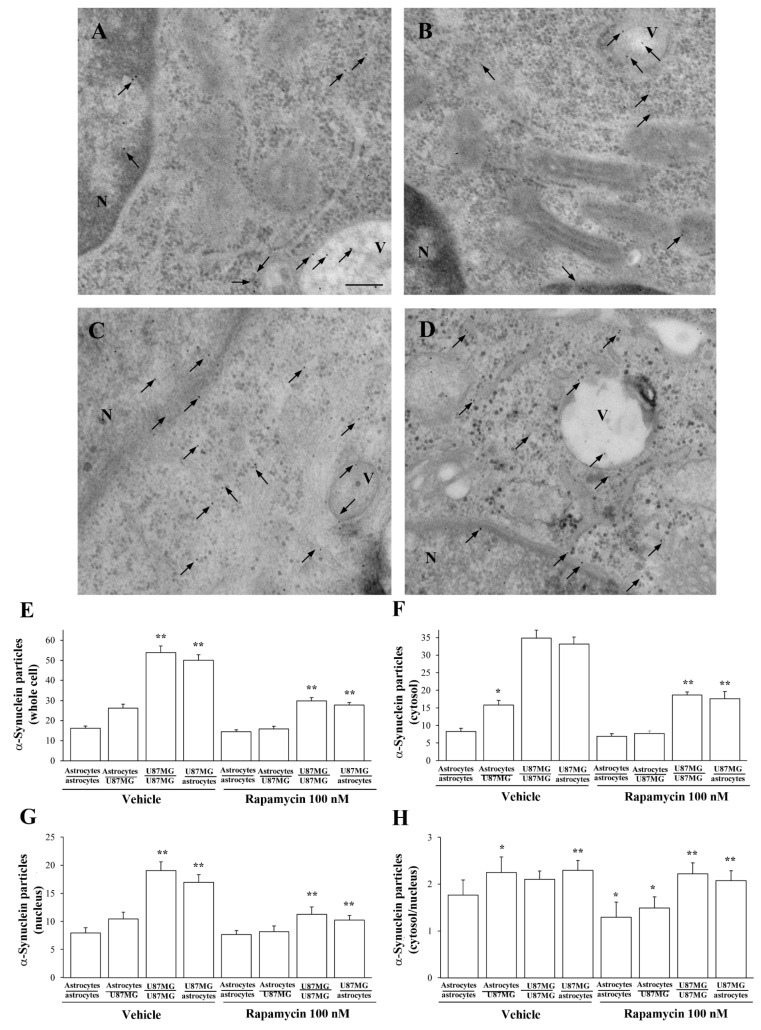
Rapamycin reduces the amount of α-syn in astrocytes co-cultured with GBM cells. Representative micrographs show α-syn immuno-gold particles within both cytosol and nucleus (black arrows) of astrocytes co-cultured with astrocytes treated with rapamycin (**A**), astrocytes co-cultured with GBM cells treated with rapamycin (**B**), GBM cells co-cultured with GBM cells treated with rapamycin (**C**), and GBM cells co-cultured with astrocytes treated with rapamycin (**D**). N = nucleus; V = vacuoles. Ultrastructural stoichiometry of α-syn immuno-gold in co-cultures of astrocytes and GBM cells, both in baseline conditions and following rapamycin administration are reported in the graphs. In detail, graphs report the number of α-syn immuno-gold-particles within whole cell (**E**), cytosol (**F**), and nucleus (**G**). The ratio of α-syn immuno-gold particles within cytosol vs. nucleus is reported in (**H**). These data are obtained from astrocytes co-cultured with astrocytes, astrocytes co-cultured with GBM cells, GBM cells co-cultured with GBM, and GBM cells co-cultured with astrocytes, both in baseline conditions and following rapamycin. Data are given as the mean *±* S.E.M. from N = 30 cells per each experimental group. * *p* < 0.05 compared with astrocytes co-cultured with astrocytes; ** *p* < 0.05 compared with astrocytes co-cultured with GBM cells. Scale bar = 0.12 μm.

**Figure 16:**
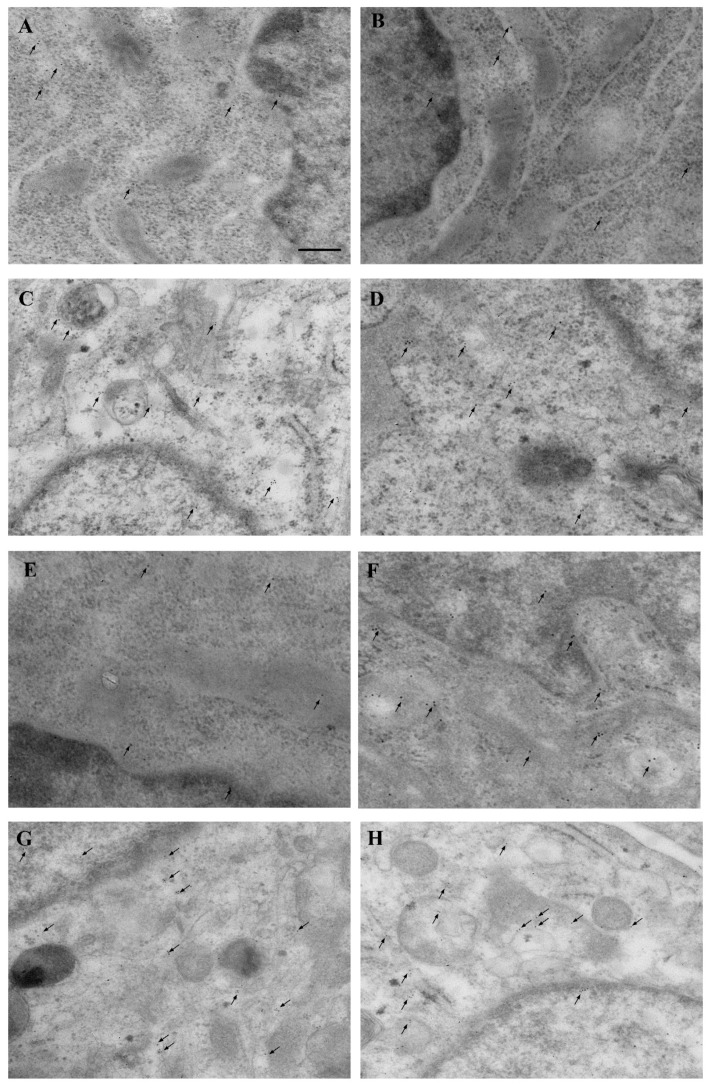
α-Syn immuno-electron microscopy in co-cultures of astrocytes and A172 cells in baseline and following rapamycin administration. Representative micrographs show α-syn immuno-gold particles (arrows) within cytoplasm and nucleus of astrocytes co-cultured with astrocytes (**A**); astrocytes co-cultured with A172 cells (**B**); A172 cells co-cultured with A172 cells (**C**), A172 cells co-cultured with astrocytes (**D**), astrocytes co-cultured with astrocytes treated with rapamycin (**E**), astrocytes co-cultured with A172 cells treated with rapamycin (**F**), A172 cells co-cultured with A172 cells treated with rapamycin (**G**), and A172 cells co-cultured with astrocytes treated with rapamycin (**H**). Scale bar = 0.15 μm.

**Figure 17:**
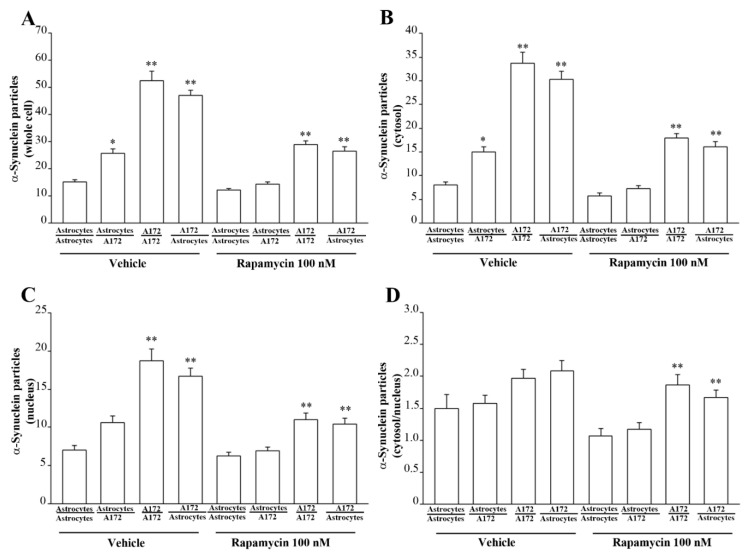
Ultrastructural stoichiometry of α-syn immuno-gold in co-cultures of astrocytes and A172 cells both in baseline conditions and following rapamycin administration. Graphs report the number of α-syn immuno-gold-particles within whole cell (**A**), cytosol (**B**), and nucleus (**C**). The ratio of α-syn immuno-gold particles within cytosol vs nucleus is reported in (**D**). These data are obtained from astrocytes co-cultured with astrocytes, astrocytes co-cultured with A172 cells, A172 cells co-cultured with A172 cells, and A172 cells co-cultured with astrocytes, both in baseline conditions and following rapamycin. Data are given as the mean ± S.E.M. from N = 30 cells per each experimental group. * *p* < 0.05 compared with astrocytes co-cultured with astrocytes; ** *p* < 0.05 compared with astrocytes co-cultured with GBM cells.

Figure 15 reports representative micrographs of α-syn immuno-cytochemistry in trans-well co-cultures of astrocytes and GBM cells following rapamycin. In detail, following rapamycin, the amount of α-syn is very low within the cytosol and nucleus of astrocytes co-cultured with astrocytes (Figure 15A) compared with that occurring within GBM cells co-cultured with GBM cells (Figure 15B), where the protein is abundant. In contrast, a marked decrease in α-syn immuno-gold is observed following rapamycin in astrocytes co-cultured with GBM cells (Figure 15C). At the same time, rapamycin significantly decreases the amount of α-syn immuno-gold particles, which occurs within GBM cells co-cultured with astrocytes (Figure 15D), which features comparable amounts of the protein compared with GBM cells co-cultured with GBM cells (Figure 15B). This is confirmed by the graphs of Figure 15, where the ultrastructural stoichiometry of the protein is reported. In detail, when counting α-syn immuno-gold particles within whole astrocytes in co-culture with GBM cell (Figure 15E), α-syn is markedly suppressed by rapamycin. In these conditions, the α-syn level is similar to that occurring within astrocytes co-cultured with astrocytes. The suppression of α-syn particles is more remarkable within the cytosol than nucleus, as reported in the graphs of Figure 15F and Figure 15G, respectively. Similar results are obtained in co-cultures of astrocytes and A172 cells, as shown in representative micrographs of Figure 16. Graphs of Figure 17 report quantitative data of α-syn as measured in co-cultures of astrocytes and A172 cells.

**Figure 18:**
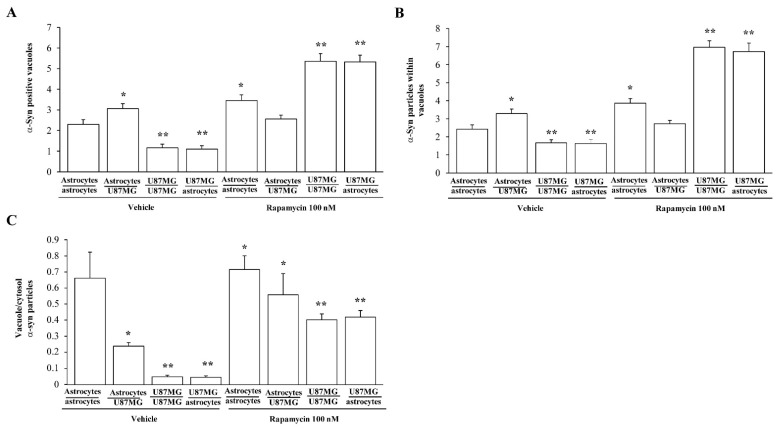
Ultrastructural stoichiometry of α-syn compartmentalization in co-cultures of astrocytes and GBM cells. The amount of α-syn positive vacuoles is reported in (**A**), while the mean number of α-syn immuno-gold particles within each vacuole is reported in (**B**). The ratio of α-syn immuno-gold particles within vacuoles vs. cytosol is reported in (**C**). These data are obtained from astrocytes co-cultured with astrocytes, astrocytes co-cultured with GBM cells, GBM cells co-cultured with GBM cells, and GBM cells co-cultured with astrocytes, both in baseline conditions and following rapamycin. Data are given as the mean *±* S.E.M. from N = 30 cells per each experimental group. * *p* < 0.05 compared with astrocytes co-cultured with astrocytes; ** *p* < 0.05 compared with astrocytes co-cultured with GBM cells.

In graphs of Figure 18, the effects of rapamycin on α-syn compartmentalization within vacuoles are specifically assessed within trans-well co-cultures by using immuno-gold counts. In detail, graphs of Figure 18 report that within astrocytes co-cultured with GBM cells a decrease in the number of α-syn immuno-positive vacuoles (graph of Figure 18A) and the number of α-syn immuno-gold particles within each vacuole (graph of Figure 18B) is measured compared with baseline astrocytes. This produces a significant decrease in the ratio between vacuolar and cytosolic amount of α-syn (Figure 18C). Such a condition is similar to that measured within GBM cells in baseline conditions. In contrast, when rapamycin is administered, it increases the number of α-syn positive vacuoles and the number of α-syn particles within vacuoles (Figure 18A and Figure 18B, respectively). This leads to an increase in the ratio between α-syn within vacuoles and α-syn within cytosol (Figure 18C). This increase in α-syn vacuole compartmentalization induced by rapamycin is similarly detected within GBM cells both co-cultured with themselves and with astrocytes (Figure 18). These phenomena concerning vacuolar placement of α-syn confirm the shift towards a GBM-like phenotype, which is induced in astrocytes co-cultured with GBM cells. Again, such a shift is reversed by a dose of rapamycin in the therapeutic range, which leads the ultrastructural compartmentalization of α-syn similar to control astrocytes.

### 3.9. Occurrence of α-Syn in the Medium and Supernatant Supernatant

**Figure 19:**
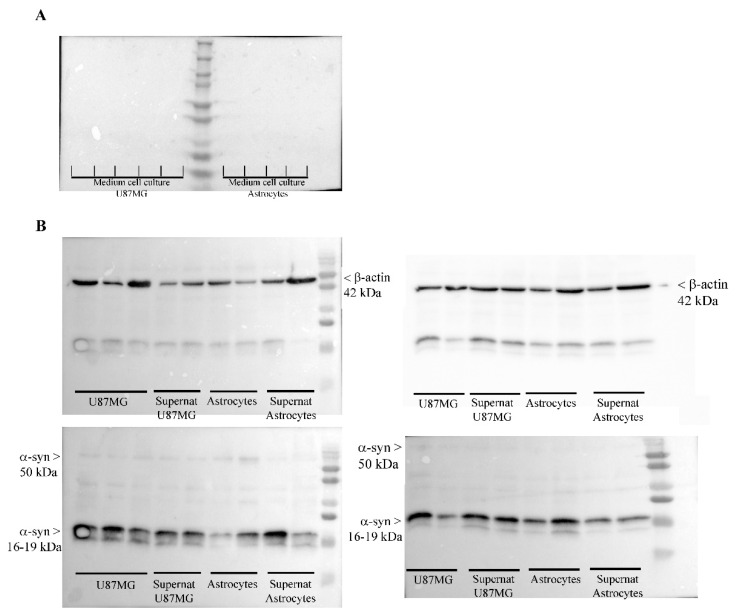
Western blotting of α-syn within cell supernatant and cell free medium. (**A**) Western blotting of the medium used for the trans-well system shows that no α-syn is detectable in the absence of either GBM cells or astrocytes, although the amount of medium being blotted corresponds to ninety-fold the amount, where a cell pellet grows. (**B**) Western blot of cell pellets and supernatant show very similar bands of α-syn. However, since the protein is blotted in a volume of supernatant, which exceeds almost ninety-fold that present in contact with the cell pellet, the real amount of α-syn detectable in the supernatant is very low compared with cell pellet. Supernat = supernatant.

**Figure 20:**
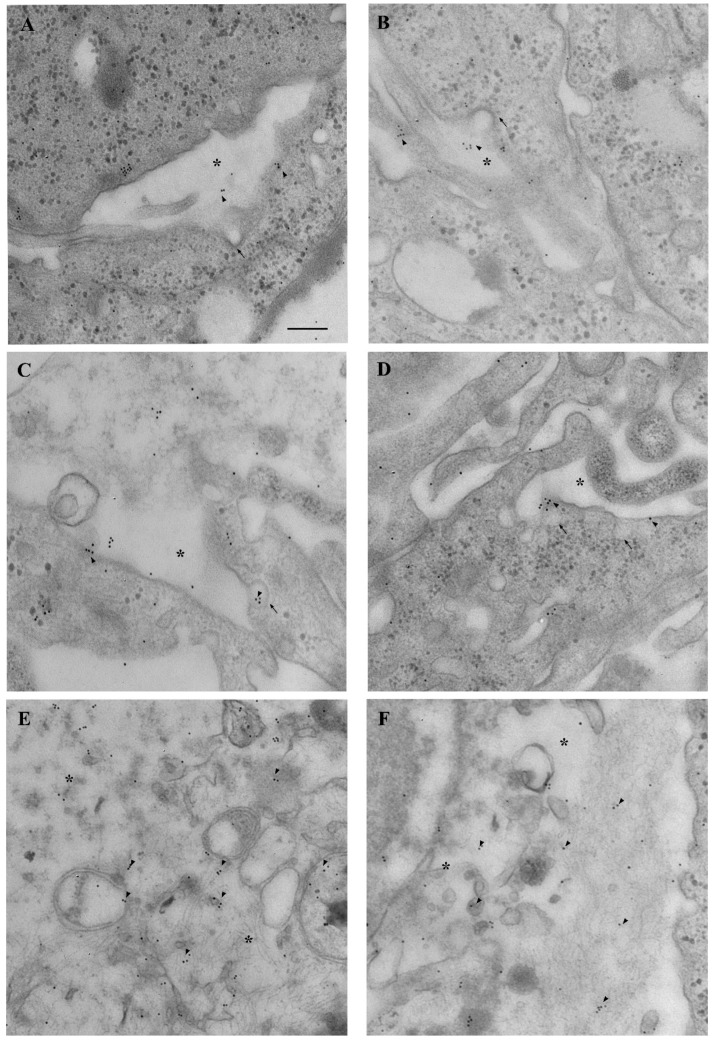
Representative in situ detection of α-syn within supernatant. Ultrastructural immuno-electron microscopy documents the presence of α-syn (arrowheads) as immuno-gold particles. These appear as free molecules (**A**), often close to exocytotic structures (arrow) (**B**) or within cell remnants containing exosome-like structures (**C**), and lying over free-floating membranes (*) (**D**–**F**). It is remarkable that these structures visualized at TEM possess a size which is compatible with the pore of the semi-permeable membrane of the trans-well system (0.4 μm). The pictures are taken after scanning the supernatant and they focus on cell membrane-rich areas. Scale bar = 0.12 μm.

Western blotting carried out in the culture medium used for the trans-well system shows that, no α-syn is detectable in the medium in the absence of GBM cells or astrocytes (Figure 19). This is confirmed even increasing the amount of the medium by sixty-six-fold the amount corresponding to a single cell pellet. Conversely, α-syn is faintly detectable in the supernatant. This amount is very low compared with the cell pellet (Figure 19). In fact, when comparing the blots of a cell pellet with supernatant similar bands of α-syn are detectable when the protein is blotted in a volume of supernatant, which exceeds almost ninety-fold the corresponding to a cell pellet. When such a ninety-fold excess of supernatant is blotted, the amount of α-syn which is measured approaches the amount measured in the cell pellet (0.85 ± 0.25 and 1.14 ± 0.09, in the supernatant and cell pellet, respectively). This is expected when considering a cell-to-cell transmission of the protein, which is present in the extracellular space in trace amount at any specific sampling time point, which remains way below the protein amount being stored permanently within the cell pellet. In fact, the amount of α-syn detected in the supernatant should be reduced by at least sixty-six-fold to be compared with the amount of α-syn detected in the cell pellet, considering volume discrepancy (as reported in the Methods). Differences between cell pellet and supernatant are even more pronounced when considering the amount of α-syn within astrocytes cell pellet (0.85 ± 0.25) and that detected within the supernatant of astrocytes (0.19 ± 0.05). This leads to a ratio pellet/supernatant = 0.75 for GBM cell cultures and a ratio pellet/supernatant = 0.22 for astrocyte cell cultures. This means a three-fold lower amount of α-syn in the supernatant outside astrocytes compared with GBM cells. To substantiate this rough amount obtained by Western blotting, a specific in situ ultra-structural investigation of α-syn immuno-gold particles was carried out in the extracellular space sampled as supernatant. In this space, TEM allows in situ identification of sporadic α-syn molecules. Several micro-shots at electron microscopy are reported in Figure 20 showing the presence α-syn as free molecules, often close to exocytotic structures or within cell remnants containing exosome-like structures and lying over free-floating membranes. It is remarkable that all these structures visualized at TEM by sampling and scanning the supernatant possess a size, which is compatible with the pore of the semi-permeable membrane of the trans-well system (0.4 μm). The occurrence of membrane remnants and cytosolic compartments within the supernatant is also witnessed by the presence of the filament-related protein β-actin, here reported as housekeeping for adjusting the optical density of α-syn (Figure 19).

## 4. Discussion

Evidence has been recently provided that within GBM cells α-syn is overexpressed compared with astrocytes [34]. Overexpression of α-syn is more marked when considering the PK-resistant isoform. Such an overexpression is sensitive to mTOR inhibition. In fact, rapamycin, through mTOR inhibition, activates autophagy, which in turn produces the clearance of highly expressed, both total, and PK-resistant α-syn [34]. In detail, the suppression of both total and PK-resistant α-syn occurs dose-dependently following rapamycin, at doses which effectively inhibit mTOR, as shown by a decrease in the enzymatic product pS6. In particular, in baseline conditions, immunoblotting of α-syn shows the presence of various α-syn bands, which are likely to represent various aggregates of α-syn molecules. These oligomeric α-syn disappears in the presence of PK, apart from one band. This suggests that PK treatment, by also digesting the binding between various α-syn molecules, may also modify the aggregation of α-syn units.

In the present study, which represents a follow-up, evidence is provided showing that high amount of α-syn detected in GBM cells may transmit to neighboring co-cultured astrocytes through a trans-well apparatus. This is characterized by a semi-permeable membrane, where pores of 0.4 μm impede cell transfer (Figure 1). The overexpression of α-syn within GBM co-cultured astrocytes occurs along with the overexpression of the stem cell marker nestin and CD133, and the shift of cell morphology and ultrastructure towards a GBM-like phenotype (GBM-primed astrocytes) (Figure 2, Figure 3, Figure 4, Figure 5, Figure 6 and Supplementary Figures S5–S8). All these phenomena are concomitantly reverted by exposure of these cells to rapamycin, which promotes differentiation of GBM cells as previously shown [19,46]. Both α-syn, nestin, and CD133, which are negligibly expressed within astrocytes in baseline conditions are abundant within U87MG, GBM cell line and they do pass from the latter to the former in the trans-well system (Figure 7, Figure 8, Figure 9, Figure 10, Figure 11, Figure 12, Figure 13, Figure 14, Figure 15, Figure 16, Figure 17, Figure 18, and Supplementary Figures S5–S8).

When the amount of nestin, CD133, and α-syn is measured in another GBM cell line, the A172 cells, similar results were obtained (Figure 10, Figure 12, Figure 16, Figure 17 and Supplementary Figures S1–S8). 

This study carried out in two different GBM cell lines calls for further and extended investigations. For instance, a specific study on astrocytoma grade I-III may clarify whether different stages of astrocytoma may express various amount of these proteins. Moreover, dedicated α-syn and nestin immuno-detection is needed in single patients to correlate the expression of α-syn with prognosis and malignancy of the tumor.

In astrocytes both α-syn and nestin expression is very low in baseline conditions (Figure 2) [47,48,49,50,51,52]. In contrast, in GBM cells a-syn and nestin are abundantly expressed (Figure 2). High expression of nestin in GBM cells identifies a sub-population of tumor-initiating cells responsible for high malignancy, chemo-resistance and relapse [53,54].

In the trans-well system, α-syn may prime co-cultured astrocytes to overexpress both α-syn and stem cell markers such as nestin and CD133. These findings are strengthened by quantitative ultrastructural stoichiometry of the protein carried out at TEM, where the amount of α-syn occurring in the cytosol, nucleus, and whole cell is calculated (Figure 4). Thus, in astrocytes co-cultured with GBM cells, the amount of α-syn within the whole cell is significantly increased to approach levels detected within GBM cells. This happens along with high expression of stem cell markers and development of a GBM-like phenotype featuring trans-cellular processes of the plasma membrane, which contrasts with plain plasma membrane of astrocytes (Figure 5).

When considering the distribution of α-syn within GBM cells, a significant amount, which exceeds 20% of cytosolic α-syn immuno-gold particles, is placed just at the level of these processes outsourcing from the plasma membrane. Such an amount is two-fold higher compared with the amount of α-syn placed on the plasma membrane of control astrocytes. It is remarkable that when astrocytes are co-cultured with GBM cells the plasma membrane develops GBM-like convolution and at this level of the plasma membrane α-syn rises up to levels similar to GBM cells (Figure 5).

This placement of α-syn mostly occurs at convoluted arrangement of the plasma membrane of GBM cells and GBM-primed astrocytes. This is consistent with the fact that α-syn stimulates membrane-bending and remodeling [55,56]. Again, α-syn is key for trans-cellular communication enabling membrane fusion, which indicates a role of α-syn in trans-cellular vesicles trafficking [57,58,59,60,61]. In keeping with this, recent evidence indicates that α-syn induces membrane thinning [62], as well as the formation of tubular structures [55,63], which in turn may physically connect cells and promote the spreading of this protein between cells [64]. Thus, it is likely that a high α-syn expression in GBM cells along with its placement within cell processes may enable cell-to-cell communication, promoting the occurrence of trans-cellular plasma membrane elongations. All these effects are likely to represent connected phenomena rather than stochastic concomitant events. 

A key point of the present study is the evidence that α-syn transmits from over-expressing GBM cells to normal astrocytes, which otherwise express negligible levels of α-syn in baseline conditions. This cell-to-cell transmission of α-syn is reminiscent of what has been described for catecholamine-containing cells during neurodegeneration [35,43,65,66,67]. Consistently, in the present study, we demonstrate that no α-syn is detectable in the culture medium in the absence of GBM/astrocyte cells (Figure 19). In contrast, some α-syn is detected in the cell supernatant, although much less compared with cell pellets (Figure 19). In fact, when considering cell-to-cell transmission of the protein, α-syn is expected to occur in the extracellular space in trace amount at each specific time point. Conversely, the cell storage of α-syn is way in excess. In fact, it is expected that only a few α-syn molecules are present in the intercellular space compared with the amount of the protein, which is present within GBM cells. This is further validated by in situ immuno-gold detection, which allows us to identify the authentic presence of intercellular α-syn molecules within exocytosis-dependent structures outgrowing from viable GBM cells (Figure 20). In the supernatant α-syn molecules are also detected as free protein or within membrane remnants which possess a size compatible with their diffusion through the semipermeable membrane of the trans-well system. The ratio of α-syn occurring in the supernatant and α-syn present in the cell is much higher in GBM cell cultures compared with astrocytes cultures. This is compatible with a more effective α-syn release from GBM cells compared with astrocytes.

The occurrence of exosome-related structures and membrane remnants in the supernatant of cell cultures, which is detected in situ by scanning the extracellular space at transmission electron microscopy, is reporting the occurrence of α-syn as immuno-gold particles associated with authentic cell-derived structures rather than a sample contamination or non-specific staining. This is confirmed by the occurrence of the cyto-filament-related protein β-actin, used here as a housekeeping, which is present in the supernatant as well. Thus, the supernatant reports both single proteins as detected by immuno-gold stoichiometry of α-syn and cell-derived structures, which appear as membrane remnants. The origin of these membrane remnants appears to be partly related to cell-to-cell communicating structures. Nonetheless it cannot be ruled out that some eventual cell damage may occur, which contributed to the occurrence of both α-syn and β-actin. In fact, one should still consider that some α-syn which is detected in the supernatant may be produced by spontaneous cell death which eventually occurs. On the other hand, the increase in α-syn produced by GBM within astrocytes co-cultured in a trans-well system may also represent the consequence of other diffusible molecules produced by GBM cells, which may induce α-syn overexpression in astrocytes. The specific molecules which may induce these effects include the PrPc, which is also overexpressed in GBM. Dedicated studies are required to analyze all these issues. In fact, the identification of α-syn within supernatant and occurrence of high α-syn level within astrocytes co-cultured with GBM cells is the first step towards identifying the scenario which may be involved in priming normal astrocytes towards a GBM-like phenotypic shift. Dedicated studies where specific molecules are conjugated with green fluorescent protein or other tagging conjugates are required, along with the study of their epigenetic effects.

The present study indicates that, either due to trans-cellular transmission or to trans-cellular induction, a net increase in α-syn within astrocytes by contiguous GBM cells is quantified by stoichiometric analysis. In detail, evidence is provided showing that α-syn-overexpressing GBM cells are able to prime co-cultured astrocytes, which in turn develop high amounts of α-syn, nestin and CD133 along with a phenotype, which is reminiscent of GBM cells. In a wider scenario, when considering the microenvironment of GBM cells in vivo, it is conceivable that beyond astrocytes, other cell types including microglia, neighboring neurons along with blood vessels may play a specific role in cell-to-cell spreading of GBM, which deserves to be investigated in the future.

The potential cell-to-cell spreading of α-syn within these GBM co-cultures is in line with recent evidence, which demonstrates how altered cell-to-cell communication of prion-like proteins may foster GBM tumor spreading, neuro-invasion, and immune escape [21]. Thus, the concept, which typically applies to cell propagation and spreading of α-syn as a prionoid in the course of neurodegenerative disorders, may apply to GBM as well, to act as an epigenetic modulator activating cancer-promoting genes. As a matter of fact, α-syn does behave as a prionoid [68,69,70,71], and upon exposure at plasma membrane it may pass from one cell to another to produce various effects on recipient cells. For instance, accumulation of α-syn may trigger a biochemical cascade, which induces cell proliferation [27,72,73,74,75,76]. In fact, α-syn promotes strong DNA methylation, which in turn triggers epigenetic effects [77]. Again, α-syn reduces histone H3 acetylation, thus affecting the expression of several genes responsible for cell survival [78,79].

This is in line with present findings showing how the increase in α-syn within GBM-primed co-cultured astrocytes is induced concomitantly with a dramatic increase in the expression of stem-like proteins, which in turn have not been simultaneously explored so far. For instance, when looking at nestin compared with α-syn, a similar increase is detected within astrocytes following co-culture with GBM cells as quantified by ultrastructural stoichiometry of the antigen. In turn, rapamycin, which brings GBM cells to cell cycle arrest, blocks cell migration, and induces cell differentiation [19,46], while inhibiting over-expression of α-syn and stem cell markers. This occurs in GBM cells and, most remarkably, within GBM primed astrocytes, which shift back towards the original glial phenotype. This evidence strongly suggests a correlation between stem-like properties, α-syn content, prionoid diffusion and cell-to-cell priming in GBM cells. 

## 5. Conclusions

In this scenario, one is tempted to hypothesize that overexpression of α-syn within GBM cells may work as a trigger to prime co-cultured naïve astrocytes towards a GBM-like phenotype, thus posing α-syn-dependent cell-to-cell communication as an unconventional mechanism for GBM proliferation and spreading.

Thus, it becomes crucial to establish which cell mechanism may induce a primary increase in α-syn independently from compensatory mechanisms. Since the amount of α-syn critically depends on the status of protein-clearing pathways and mostly autophagy [20], an emphasis should be posed on which condition features autophagy suppression. This is the case of GBM cells, which are characterized by a marked over-activation of mTOR, which in turn suppresses autophagy, while contributing to GBM cell proliferation and stemness [13,15,17,18]. On the other hand, previous studies, in line with the present report, demonstrate that inhibition of autophagy increases α-syn secretion and cell-to-cell transfer [80,81,82].

When mTOR overactivity is suppressed by the mTOR inhibitor rapamycin we could measure a decrease in α-syn, which is associated with an increased number of autophagy vacuoles. In fact, while a decrease in alpha-synuclein occurs, a higher amount of residual alpha-synuclein is placed within LC3-positive autophagy vacuoles.

Altogether, these findings suggest a novel marker for GBM cells, which accompanies classic stem cell markers, and pose the significance of α-syn accumulation beyond a hallmark of degenerative disorders to include rapidly progressing neuro-oncological conditions such as GBM.

## Data Availability

The data that support the findings of this study are available from the corresponding author upon reasonable request.

## Supplementary Materials

The following supporting information can be downloaded at: https://www.mdpi.com/article/10.3390/cancers14061417/s1, Figure S1: Rapamycin dose-dependently reduces a-syn immuno-fluorescence in A172 cells, Figure S2: Rapamycin reduces a-syn assessed by immuno-blotting in A172 cells, Figure S3: Rapamycin dose-dependently reduces a-syn and nestin immuno-fluorescence in A172 cells, Figure S4: Rapamycin dose-dependently reduces a-syn and nestin immuno-fluorescence in A172 cells, Figure S5: Rapamycin dose-dependently reduces CD133 immunostaining in U87MG cells, Figure S6: Rapamycin dose-dependently reduces CD133 immunostaining in A172 cells, Figure S7: CD133 immuno-cytochemistry in co-cultures of astrocytes and U87MG cells in baseline and following rapamycin administration, Figure S8: CD133 immuno-cytochemistry in co-cultures of astrocytes and A172 cells in baseline and following rapamycin administration.
