# Supplementary material for: Spreading of Alpha Synuclein from Glioblastoma Cells towards Astrocytes Correlates with Stem-like Properties
Source: Cancers (Basel). 2022 Mar 10;14(6):1417. doi: 10.3390/cancers14061417 (PMC8946011; doi:10.3390/cancers14061417)
Supplement: Supplementary file 1 [file cancers-14-01417-s001.zip › cancers-1573419-supplementary.pdf]

cancers

MDPI

Spreading of Alpha Synuclein from Glioblastoma Cells towards Astrocytes Correlates with Stem-like Properties

Larisa Ryskalin, Francesca Biagioni, Gabriele Morucci, Carla L. Busceti, Alessandro Frati, Stefano Puglisi-Allegra, Michela Ferrucci and Francesco Fornai

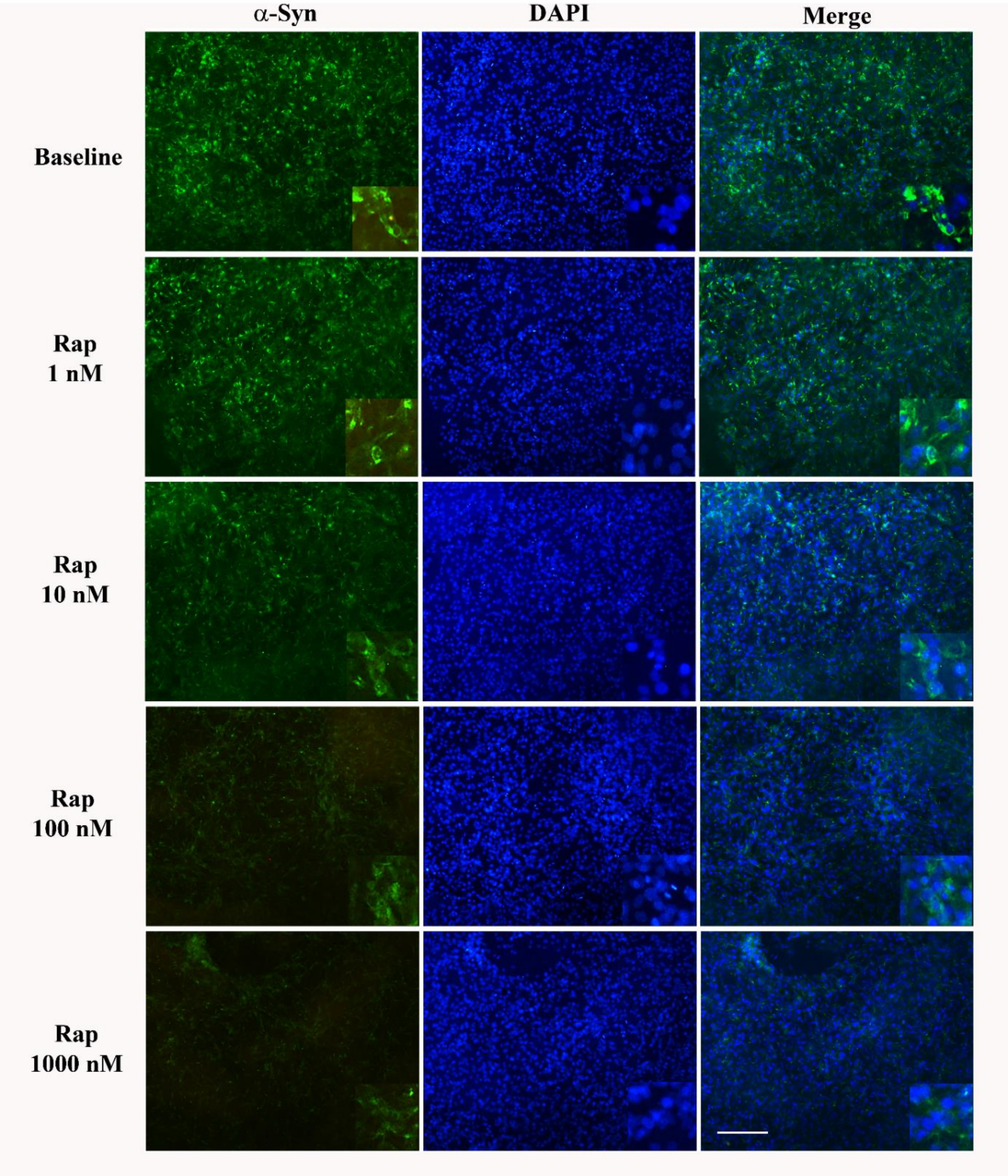

Figure S1. Rapamycin dose-dependently reduces α-syn immuno-fluorescence in A172 cells. Representative pictures of α-syn-positive A172 cells, in baseline conditions and following rapamycin

(Rap) administration. Each cell nucleus is visualized by using DAPI (blue). In these cells, the amount of α-syn is stained by a green fluorescent labeled secondary antibody (Alexa 488, green). Scale bar=200 μm (low magnification); 50 μm (inserts).

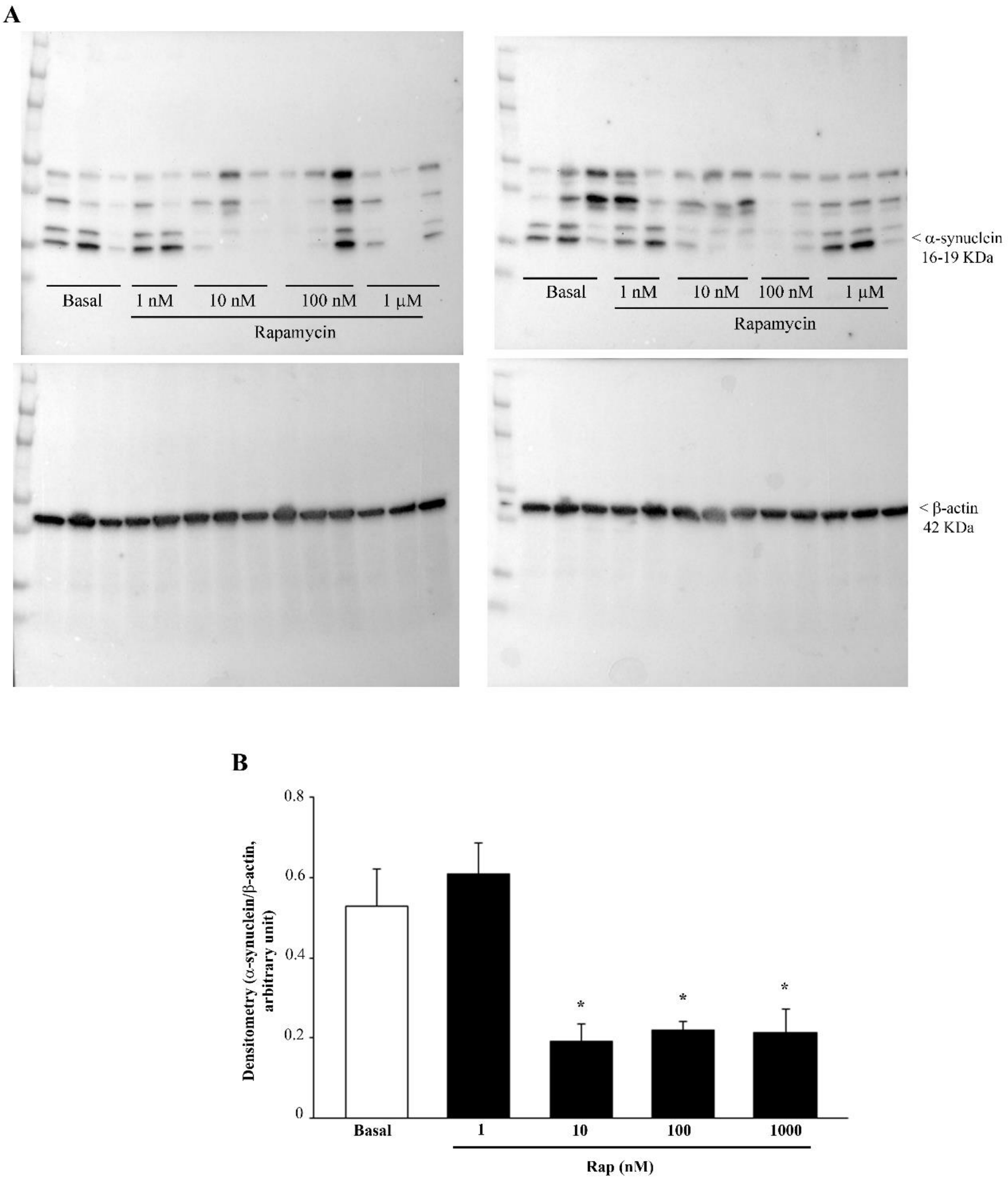

Figure S2. Rapamycin reduces α-syn assessed by immuno-blotting in A172 cells. (**A**) Representative immuno-blotting for α-syn and the housekeeping β-actin in baseline conditions (basal) and rapamycin-treated A172 cells. (**B**) The ratio between the optical densities of α-syn and β-actin is reported in the graph. Data are given as the mean±S.E.M. of the optical density for each experimental group measured in 4 up to 6 blots. *P<0.05 compared with basal.

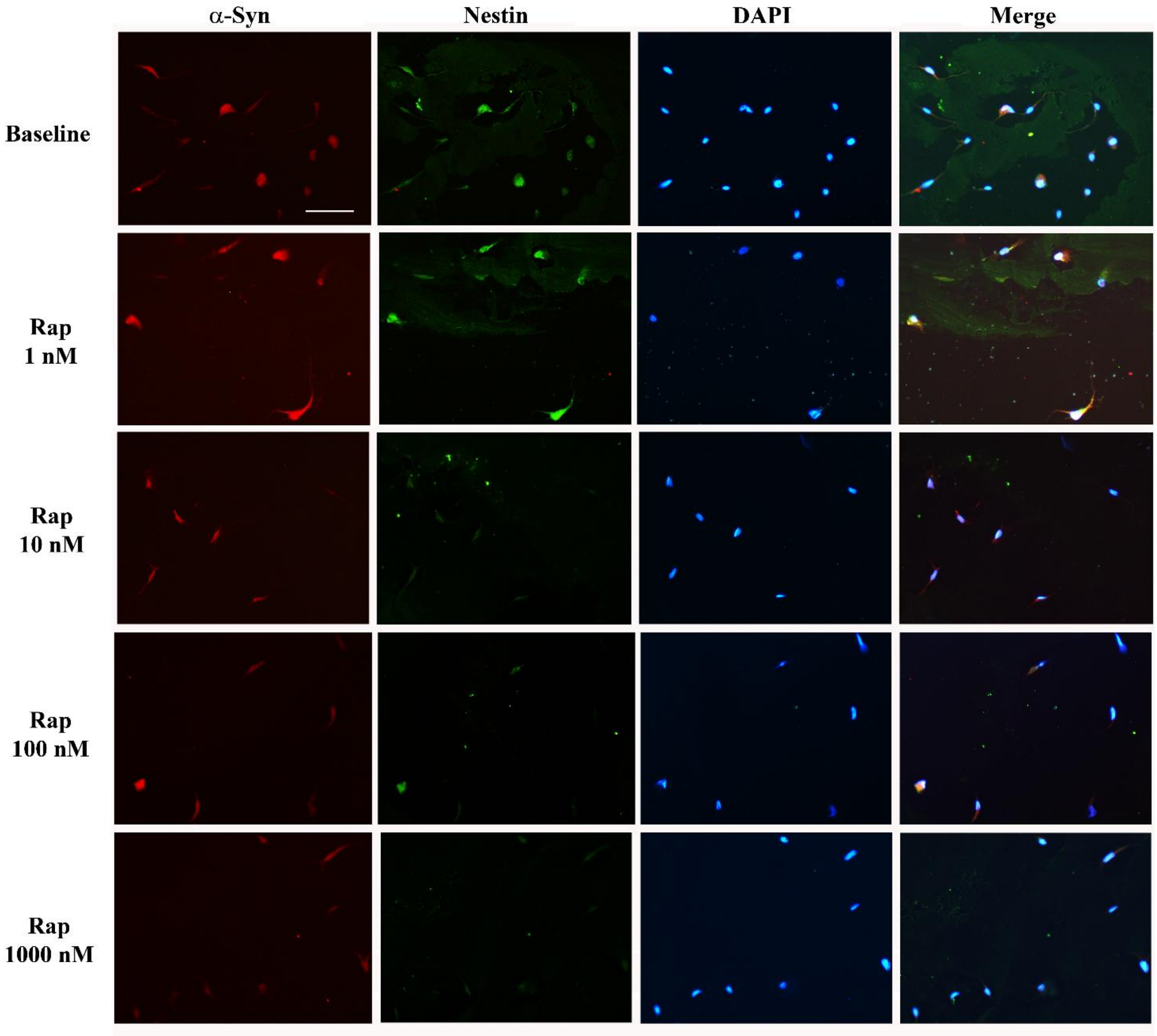

Figure S3. Rapamycin dose-dependently reduces α-syn and nestin immuno-fluorescence in A172 cells. Representative pictures show immuno-fluorescence for α-syn (red) and nestin (green) within A172 cells, both in baseline conditions and following rapamycin administration. Each cell nucleus is visualized by using DAPI (blue). Scale bar=120 µm.

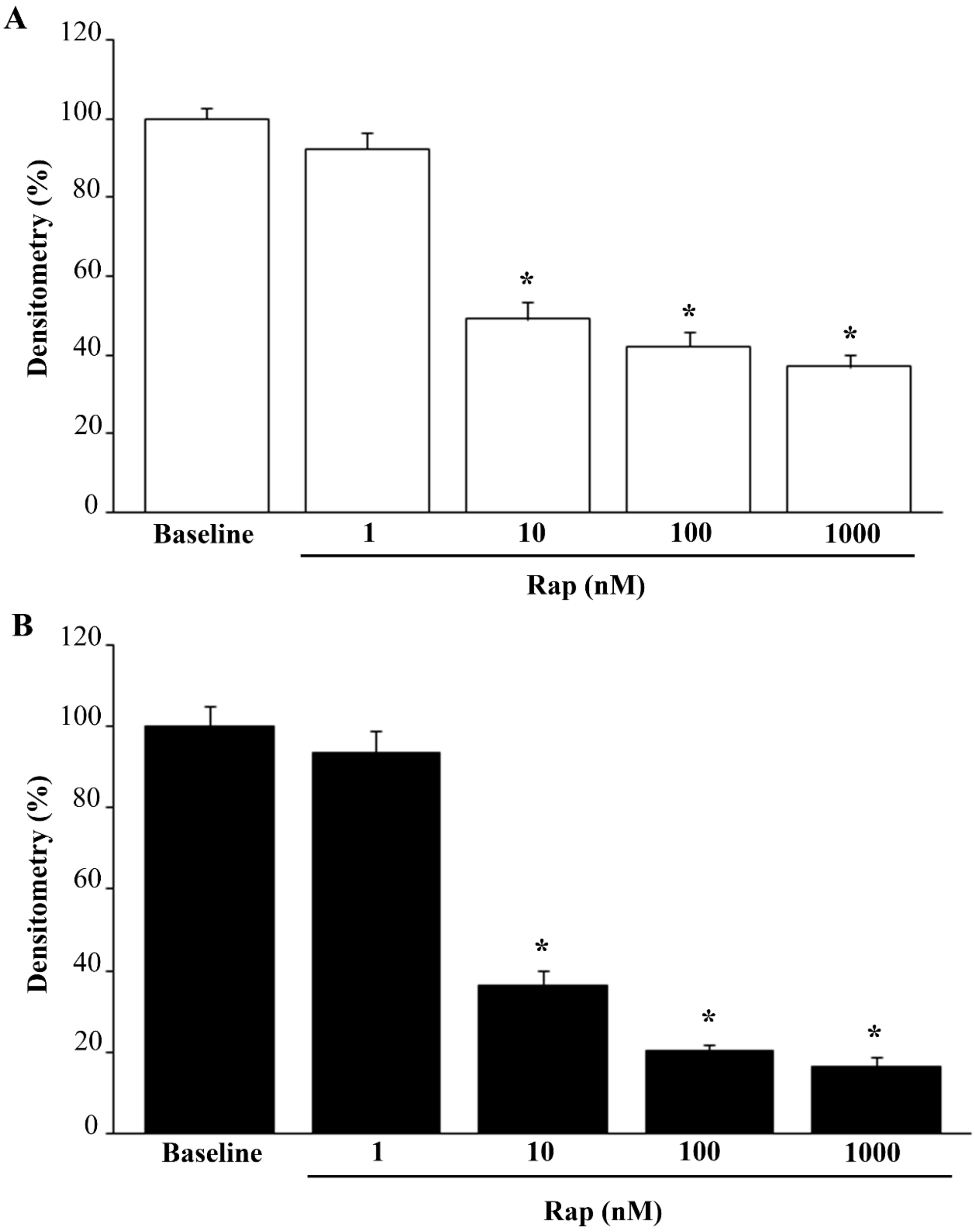

Figure S4. Rapamycin dose-dependently reduces α-syn and nestin immuno-fluorescence in A172 cells. Graphs report densitometry for α-syn (**A**) and nestin (**B**) immuno-fluorescence within A172 cells, both in baseline conditions (baseline) and following various doses of rapamycin (Rap). Data are given as the mean percentage+S.E.M. of optical density for each experimental group (assuming baseline as 100%) obtained from N=30 cells per group. *P<0.05 compared with baseline.

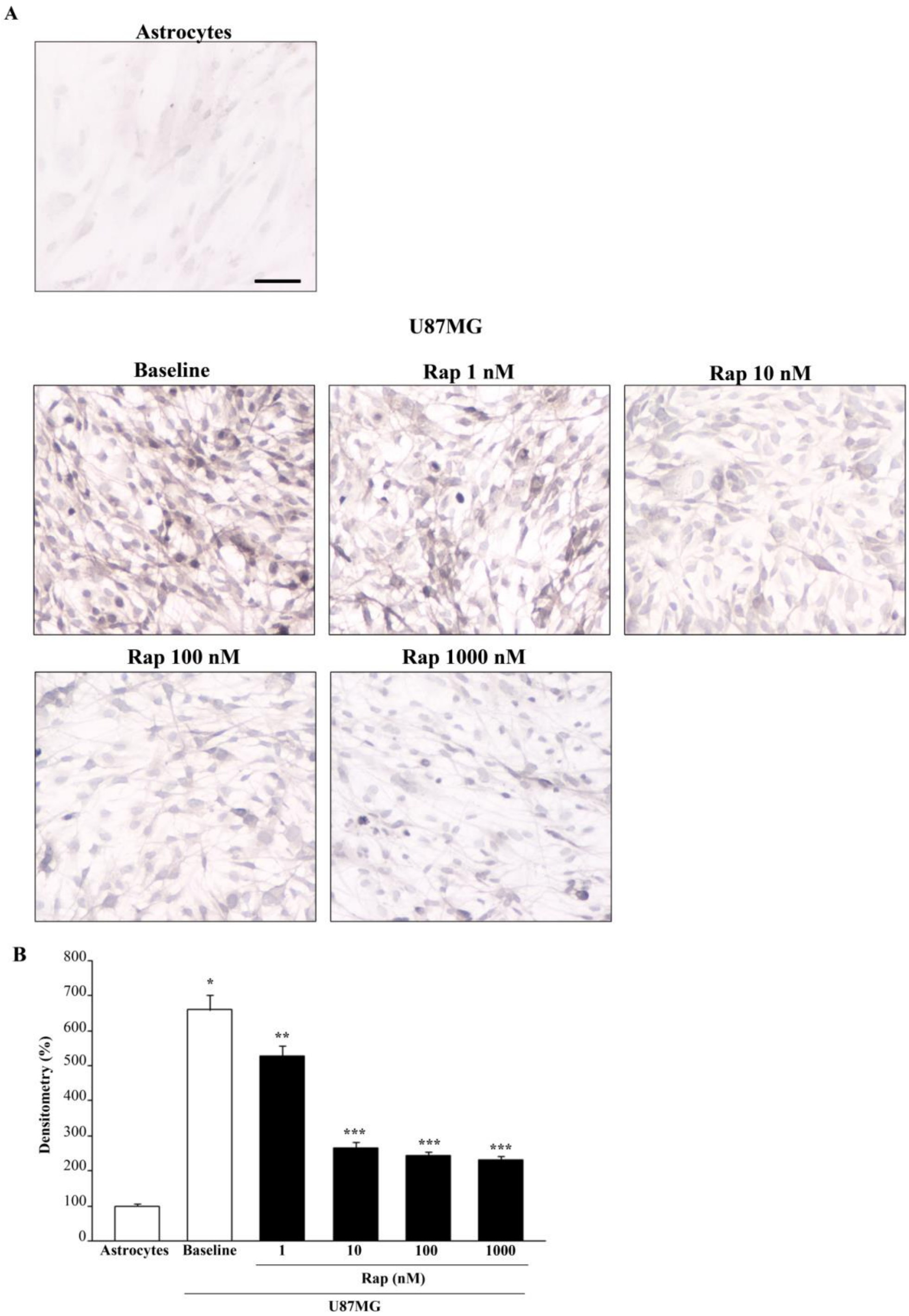

Figure S5. Rapamycin dose-dependently reduces CD133 immunostaining in U87MG cells. (**A**) Representative pictures of CD133-positive astrocytes andU87MG cells, in baseline conditions, and following rapamycin (Rap) administration. (**B**) The graph reports densitometry measured both in baseline conditions (baseline) and following various doses of rapamycin (Rap). Data are given as the mean percentage+S.E.M. of optical density for each experimental group (assuming astrocytes as 100%) obtained from N=30 cells per group. *P<0.05 compared with astrocytes; **P<0.05 compared with astrocytes and U87MG in baseline conditions; ***P<0.05 compared with astrocytes and U87MG at baseline and following Rap 1nM. Scale bar=55 μm.

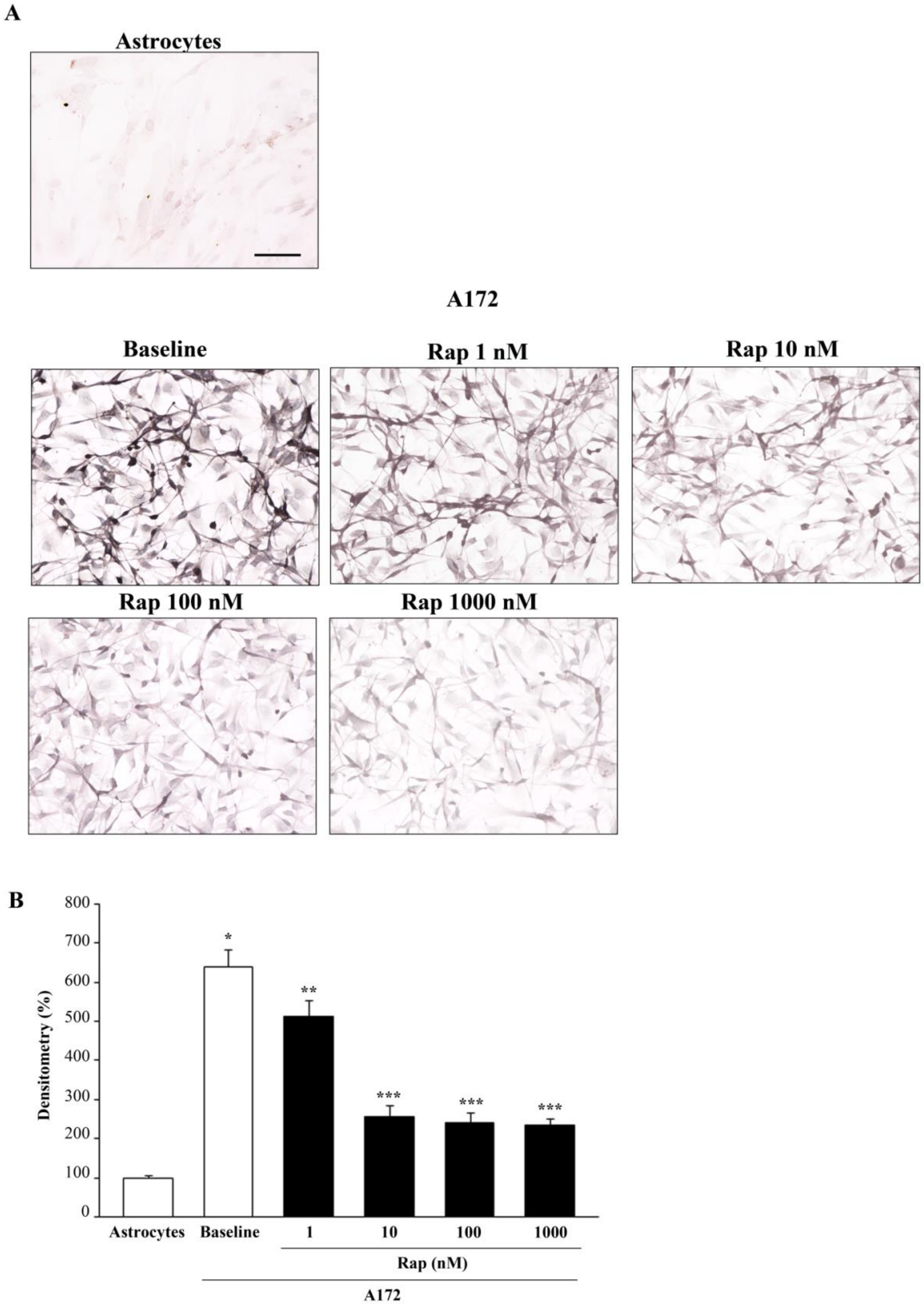

Figure S6. Rapamycin dose-dependently reduces CD133 immunostaining in A172 cells. (**A**) Representative pictures of CD133-positive astrocytes and A172 cells, in baseline conditions, and following rapamycin (Rap) administration. (**B**) The graph reports densitometry measured both in baseline conditions (baseline) and following various doses of rapamycin (Rap). Data are given as the mean percentage+S.E.M. of optical density for each experimental group (assuming astrocytes as 100%) obtained from N=30 cells per group. *P<0.05 compared with astrocytes; **P<0.05 compared with astrocytes and A172 cells in baseline conditions; ***P<0.05 compared with astrocytes, and A172 cells at baseline and following Rap 1nM. Scale bar=55 µm.

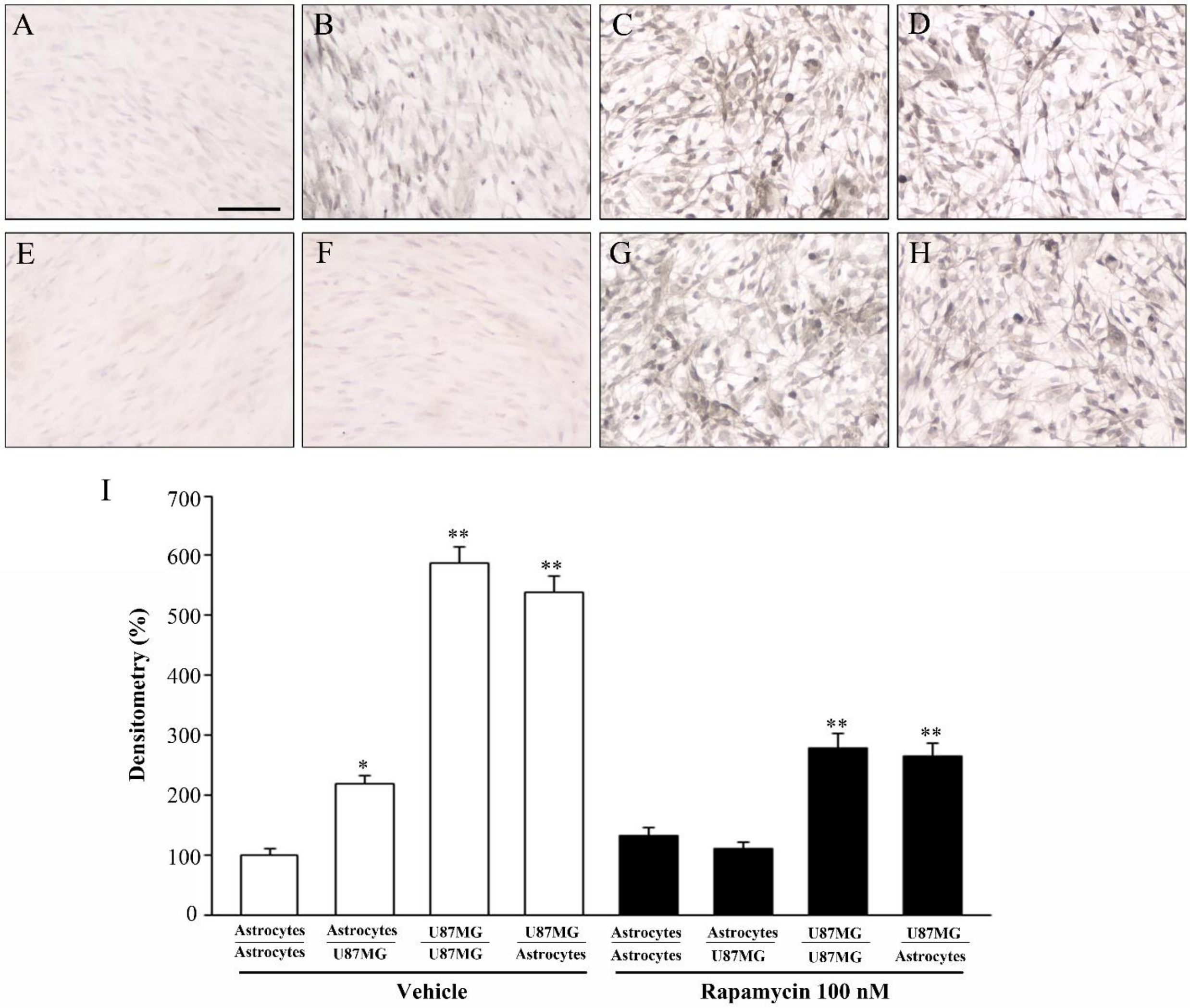

Figure S7. CD133 immuno-cytochemistry in co-cultures of astrocytes and U87MG cells in baseline and following rapamycin administration**.** Upper panels report representative pictures of CD133 immuno-cytochemistry within astrocytes co-cultured with astrocytes (**A**), astrocytes co-cultured with U87MG cells (**B**), U87MG cells co-cultured with U87MG cells (**C**), and U87MG cells co-cultured with astrocytes (**D**) in baseline conditions. Lower panels report representative pictures of CD133 immuno-cytochemistry within astrocytes co-cultured with astrocytes treated with rapamycin (**E**), astrocytes co-cultured with U87MG cells treated with rapamycin (**F**), U87MG cells co-cultured with U87MG cells treated with rapamycin (**G**), and U87MG cells co-cultured with astrocytes treated with rapamycin (**H**). (**I**) Graph reports densitometry of CD133 immuno-staining measured in co-cultures of astrocytes and U87MG cells, both in baseline conditions (vehicle) and following rapamycin administration. Data are given as the mean percentage+S.E.M. of optical density for each experimental group (assuming astrocytes co-cultured with astrocytes as 100%) obtained from N=30 cells per group. *P<0.05 compared with astrocytes co-cultured with astrocytes; **P<0.05 compared with astrocytes co-cultured with GBM cells. Scale bar=60 µm.

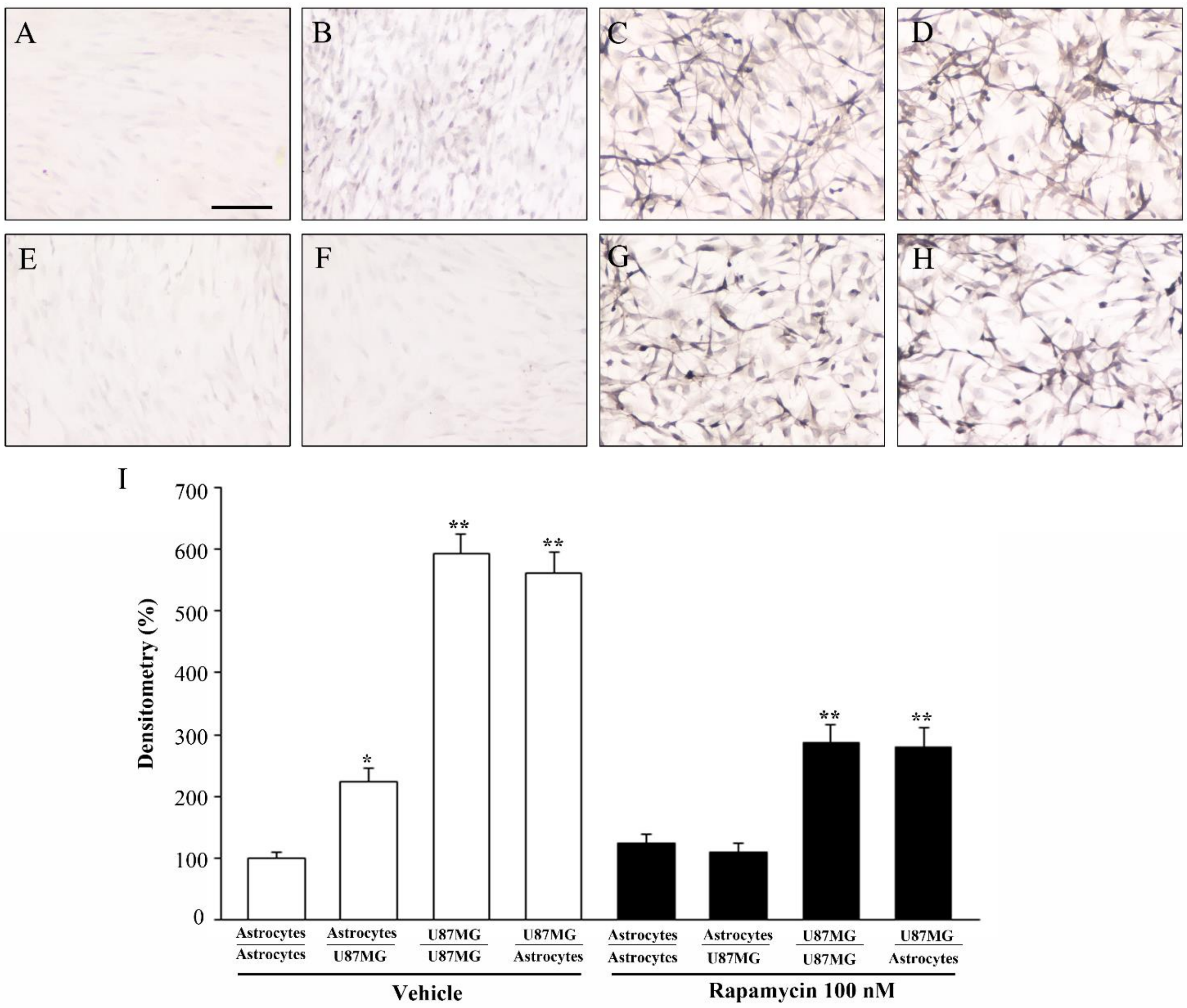

Figure S8. CD133 immuno-cytochemistry in co-cultures of astrocytes and A172 cells in baseline and following rapamycin administration. Upper panels report representative pictures of CD133 immuno-cytochemistry within astrocytes co-cultured with astrocytes (**A**), astrocytes co-cultured with A172 cells (**B**), A172 cells co-cultured with A172 cells (**C**), and A172 cells co-cultured with astrocytes (**D**) in baseline conditions. Lower panels report representative pictures of CD133 immuno-cytochemistry within astrocytes co-cultured with astrocytes treated with rapamycin (**E**), astrocytes co-cultured with A172 cells treated with rapamycin (**F**), A172 cells co-cultured with A172 cells treated with rapamycin (**G**), and A172 cells co-cultured with astrocytes treated with rapamycin (**H**). (**I**) Graph reports densitometry of CD133 immuno-staining measured in co-cultures of astrocytes and A172 cells, both in baseline conditions (vehicle) and following rapamycin administration. Data are given as the mean percentage+S.E.M. of optical density for each experimental group (assuming astrocytes co-cultured with astrocytes as 100%) obtained from N=30 cells per group. *P<0.05 compared with astrocytes co-cultured with astrocytes; **P<0.05 compared with astrocytes co-cultured with GBM cells. Scale bar=60 μm.
